# Investigating the Role of Gene-Gene Interactions in TB Susceptibility

**DOI:** 10.1371/journal.pone.0123970

**Published:** 2015-04-28

**Authors:** Michelle Daya, Lize van der Merwe, Paul D. van Helden, Marlo Möller, Eileen G. Hoal

**Affiliations:** SA MRC Centre for TB Research, DST/NRF Centre of Excellence for Biomedical Tuberculosis Research, Division of Molecular Biology and Human Genetics, Faculty of Medicine and Health Sciences, Stellenbosch University, Cape Town, South Africa; Universitat Pompeu Fabra, SPAIN

## Abstract

Tuberculosis (TB) is the second leading cause of mortality from infectious disease worldwide. One of the factors involved in developing disease is the genetics of the host, yet the field of TB susceptibility genetics has not yielded the answers that were expected. A commonly posited explanation for the missing heritability of complex disease is gene-gene interactions, also referred to as epistasis. In this study we investigate the role of gene-gene interactions in genetic susceptibility to TB using a cohort recruited from a high TB incidence community from Cape Town, South Africa. Our discovery data set incorporates genotypes from a large a number of candidate gene studies as well as genome-wide data. After limiting our search space to pairs of putative TB susceptibility genes, as well as pairs of genes that have been curated in online databases as potential interactors, we use statistical modelling to identify pairs of interacting SNPs. We attempt to validate the top models identified in our discovery data set using an independent genome-wide TB case-control data set from The Gambia. A number of models were successfully validated, indicating that interplay between the *NRG1 - NRG3, GRIK1 - GRIK3* and *IL23R - ATG4C* gene pairs may modify susceptibility to TB. Gene pairs involved in the NF-κB pathway were also identified in the discovery data set (*SFTPD - NOD2, ISG15 - TLR8* and *NLRC5 - IL12RB1*), but could not be tested in the Gambian study group due to lack of overlapping data.

## Introduction

Tuberculosis (TB) is a serious global health problem, with 8.6 million new infections and 1.2 million deaths reported in 2012 [1]. In South Africa, it is the fourth leading cause of mortality [2]. The South African Coloured population (SAC) is the largest demographic in metropolitan areas of Cape Town that have some of the highest reported incidences of TB worldwide, despite extensive BCG vaccination and low HIV prevalence [3].

Although up to a third of the world’s population has latent TB infection [1], only about 10% of immunocompetent individuals progress to disease. Many studies have established that host genetic factors are involved in the disease [4]. As is the case for other complex diseases, only a small proportion of the posited heritability has been found [5–7]. The results of TB association studies are furthermore often inconsistent between studies [8, 9].

One of the common explanations for the missing heritability of complex disease is gene-gene interactions, a.k.a. epistasis [5, 10–12]. It has also been postulated that failure to validate genetic associations in independent studies may be ascribed to epistasis [13]. Epistasis can be defined as the effect of a genetic locus on a phenotype being modified by one or more other loci. The term was first used by Bateson based on his experiments with flower color in pea plants, showing that the effects of one gene can be masked by another gene [14, 15]. A similar term, “epistacy”, was later coined by Fisher, referring to the interaction term in regression models that attempts to encapsulate the relationship between two genetic loci and an outcome variable [16]. Based on context, the term epistasis can thus either refer to biological interaction, where effects are mutually dependent and describe a state of nature, or statistical interaction, alluding to the interaction term of two or more variables in a regression model [12, 16, 17]. The notion of biological interaction has often been demonstrated experimentally in model organisms such as yeast, bacteria and animal models [11, 18–27], but this has been less successfully demonstrated in humans [26, 28]. It should also be noted that absence of detectable statistical interaction does not necessarily imply lack of biological interaction [12, 17].

The immune system is complex and comprises many intricate elements, thus progression to active TB may be elucidated by identifying the interplay of gene products in the host defence against TB infection. Only a small number of TB susceptibility gene-gene interaction studies have been published to date [29–35] and these were limited to a small number of candidate genes. In this study, we use a large sample bank of TB case and control samples collected from SAC individuals residing in areas of high TB incidence to detect gene-gene interactions that may underlie TB susceptibility. The data set constitutes genotype data collected from a large number of candidate gene studies (76 genes and 214 SNPs), as well as a large micro-array (chip) data set (388 654 SNPs, 642 cases and 91 controls). We also incorporate correction for ancestry.

A large variety of software packages have been developed to detect gene-gene interactions [36–52]. Approaches implemented in these and other packages can be be broadly classed as “traditional” regression based approaches, Bayesian frameworks, testing for allelic association, machine learning and pathway or network based approaches. In traditional regression based methods, interactions are identified by a linear model with phenotype as outcome variable and genotypes as predictor variables. These models includes interaction term(s) which measures the departure of two or more loci from additivity. Interaction models can also be identified using Bayesian frameworks. A prior distribution for the unknown parameter(s), such as the number of predictors to use in a regression model, or the type of effect markers have on the phenotype (no, main or interaction) is specified. The posterior distribution of the parameter(s) is then estimated using simulation techniques such as Markov chain Monte Carlo (MCMC). Due to the large dimensionality of especially genome-wide data sets, an initial filtering step is sometimes employed prior to testing for association using regression. A simple technique employed initially was one proposed by Marchini et al. [53], where tests for interaction are limited to loci that are marginally associated with the phenotype. A more recent strategy is to limit association testing to loci based on curated biological knowledge [54–57]. Another popular approach is the use of test statistics that can be computed efficiently [36, 52]. A particularly intuitive test statistic that measures interaction and that can be computed efficiently is a test for allelic association [48], which can be computed in cases only, or used to test for differing allelic association between cases and controls. Machine learning and data mining techniques use computationally efficient algorithms to identify a set of variables that can be used to predict or classify an outcome. These techniques are especially useful for identifying multiple predictors, and often use the notion of training and testing data sets to first train and then test models on different subsets of data. Pathway and network based approaches have also recently become popular, and describes complex networks of interactions that may affect a phenotype [26, 38, 41]. Graph theory is used to find subnetworks of genes that represent a common pathway and that are enriched for association with the outcome of interest. In this way underlying disease pathways are identified, rather than specific variants that may be interacting.

With the advent of new genome editing technologies such as CRISPR, detecting pairs of SNPs rather than pathways or networks lends itself to experimental validation. Given the size of our study group, limiting our search to pairs of SNPs only is also appropriate. Our SAC case-control data set comprises sample sets that were genotyped in a number of different studies which did not always overlap well, resulting in a relatively sparse data set. Age, gender and ancestry also differ between cases and controls [58]. We therefore used statistical modelling rather than data mining techniques to identify interactions between pairs of SNPs. Statistical modelling allows for the adjustment of known confounders, and utilizes all available data for each test, without requiring imputation or other complex strategies to deal with missing data. We also limit our search space to pairs of genes that have been identified as TB susceptibility candidate genes, and pairs of genes that have been curated in online databases as potential interactors, a strategy that has previously been used successfully [55, 56]. Finally we attempt to validate our findings in an independent Gambian TB case-control data set.

## Subjects and Methods

### Sample Collection and Ethical Approval

Individuals from the Ravensmead and Uitsig suburbs in Cape Town, who self-identified as South African Coloured, were recruited to participate in this study. (The collective term for people of mixed ancestry in southern Africa is “Coloured” and is recognized and used officially in South Africa. Whilst we acknowledge that in some cultures this term may have acquired a derogatory connotation, this is certainly not intended here.) These suburbs have a homogenous socio-economic environment, low prevalence of HIV and high incidence of TB [3]. TB patients were diagnosed using bacterial confirmation (smear positive/culture positive). Healthy individuals with no prior history of TB were selected as controls. All participants were HIV negative. Our previous study of healthy children and young adults from the control community found that 80% of children older than 15 years had positive tuberculin skin tests (TST), an indication of latent infection with *Mycobacterium tuberculosis* (*M. tuberculosis*) [59]. The majority of the control population is therefore TST positive, and with the average age of the controls in this study being 31 years, we estimate a TST positivity of 80% or above. These healthy individuals had no previous history of TB disease or treatment and were unrelated to all others included in the study.

This study was approved by the Ethics Committee of the Faculty of Health Sciences, Stellenbosch University (project registration numbers 95/072, NO6/07/132 and N11/07/210). Blood samples for DNA were collected with written informed consent, and written informed consent was obtained from the next of kin, caretakers, or guardians on behalf of the minors enrolled in the study. The research was conducted according to the principles expressed in the Declaration of Helsinki.

Sample collection and ethical approval of a Gambian tuberculosis study group, obtained from the Welcome Trust Case Control Consortium (WTCCC), are described by Thye et al. (2012, Supplementary Information) [60].

### Genotyping and Quality Control

A total of 955 case and 521 control SAC samples were collected between 1994 and 2007. The samples were used to perform a number of candidate gene studies, using unrelated individuals, summarized in S1 Table. Single nucleotide polymorphisms (SNPs) that were genotyped in these studies were used in the present study, and SNPs with a minor allele frequency (MAF) lower than 0.01 and a Hardy Weinberg equilibrium (HWE) p-value (exact test) lower than 0.01 were discarded, leaving 214 SNPs from 76 genes. The SAC sample bank was also used to genotype 969 samples on the Affymetrix GeneChip Human Mapping 500K Array set (Affymetrix 500K chip set). A total of 642 cases, 91 controls and 388 654 SNPs was retained in the data set after SNP calling [61], quality control and removal of related individuals [62]. The data set was also aligned to the Genome Reference Consortium Human genome build 37 (GRCh37). The SAC candidate gene and Affymetrix data sets were then combined, and used to identify pairs of SNPs that jointly modify the odds of having TB.

The Gambian tuberculosis data set was used to validate the top interaction models found in the SAC data set. A total of 1 498 cases and 1 496 controls was genotyped on the Affymetrix 500K chip set (more detail can be found in Thye et al., 2010, Supplementary Methods [63]). After SNP quality control (removal of SNPs with calling probability < 0.95, HWE p-value < 0.0001, MAF < 0.01, missing rate > 0.05) and alignment to GRCh37, 402 856 SNPs remained in the data set. Individuals with excess heterozygosity, outlying individuals and related individuals (degree of relatedness ≤ 2, according to identity by state estimates) were also removed. The final data set were composed of 1 156 cases and 1 206 controls.

### Limiting the Search Space based on Biological Evidence

We limited tests for interaction to SNP pairs of genes that have been identified as TB susceptibility candidate genes (which we refer to as candidate gene SNP pairs), and pairs of genes that are known to interact based on experimental evidence, or that are found in the same biological pathway, ontological category or protein family (which we refer to as biofilter SNP pairs, after the software program used to identify the pairs). A total of 76 candidate genes previously genotyped by our group, as well as 33 additional tuberculosis and pulmonary tuberculosis candidate genes curated in the HGV&TB database based on literature reviews (http://genome.igib.res.in/cgi-bin/hgvtb/inter.cgi), with at least one SNP genotyped in the SAC chip data, was used to generate 5 886 candidate gene-gene pairs, composed of 1 278 unique candidate gene SNPs. Seventeen of these SNPs were genotyped on both the Affymetrix chip and another platform, and the strand orientation of 10 of these SNPs were flipped when combining the data sets. Duplicate genotypes were available for 4 686 SNPs, of which 280 genotypes mismatched (error rate of 0.06). The mismatched genotypes were discarded. Another 2 438 interacting gene-gene pairs were identified, comprised of 28 936 unique SNPs. After discarding SNP pairs with less than 60 genotypes available for either cases or controls, 854 703 candidate gene SNP pairs and 1 040 161 biofilter SNP pairs were identified for testing.

### Statistical Analyses

Logistic regression was used to identify pairs of SNPs that jointly modify the odds of having TB. The genotypes of SNPs were encoded as factor variables, and SNPs on chromosome X of male individuals were encoded as homozygotes. Covariates, the main effects of each SNP and an interaction term were included in each model (Case/Control ∼ Covariates + SNP1 + SNP2 + SNP1×SNP2).

The p-value of the interaction term was used to detect and report the significance of interactions (4 degrees of freedom test). Reported p-values were not corrected for multiple testing. To aid interpretation of the results, the nature of the association is illustrated by graphs of the observed genotype combination proportions in the data, as interaction effects such as odds ratios are difficult to describe and interpret. Furthermore, reliable estimates of odds ratios could often not be calculated, as some of the genotype combinations include zero counts. Graphs of allele combination frequencies in cases and controls are also provided. An expectation-maximisation (EM) algorithm was used to infer allele combinations per subject. The particular algorithm was originally designed to infer haplotypes, but does not assume the physical coupling of SNPs, and is therefore also appropriate for estimating allele combination frequencies. We note that the only uncertainty in inferring these allele pairs is double heterozygotes. The logits of the possible genotype combinations are also illustrated. This demonstrates the differing direction or magnitude that a SNP has on the odds of having disease, depending on the genotype of the second SNP; non-parallel lines being indicative of an interaction effect. The effects were estimated by absorbing the marginal effects of the SNPs into the SNP × SNP interaction term, and adjusting for the covariates included in the model by averaging over them [64].

After the top interacting pairs of SNPs were identified, the individual effects of each of the identified SNPs were tested separately in the SAC and Gambian cohorts using logistic regression. SNPs were encoded as factor variables and covariates were included in each of the models (Case/Control ∼ Covariates + SNP).

Allelic interaction of the identified top SNP pairs was also tested in the SAC cohort. SNPs were encoded as numeric variables, according to the number of copies of the rare variant, as follows: 0, 1 or 2 copies of the rare variant for additive encoding, 0 or 1 for dominant encoding, with 1 representing heterozygotes and rare homozygotes, and 0 or 1 for recessive encoding, with 1 representing rare homozygotes. Each of the nine possible allelic encoding combinations were then tested for each of the identified top SNP pairs.

Age and gender are differentially distributed in the SAC TB cases and controls and gender is differentially distributed between the Gambian TB cases and controls (S2 Table, age not available for the Gambian data). Age and gender were therefore included as covariates in the SAC study group models, and gender was included as a covariate in the Gambian study group models.

Previous work has shown that TB cases have a higher proportion of African ancestry compared to controls in the SAC study group [58, 62], necessitating adjustment for ancestry. Ancestry proportions for each of the 5 source ancestries of the SAC (African San, African non-San, European, South Asian and East Asian) were estimated using a panel of 116 AIMs, as described previously [58]. Ancestry proportions were estimated in a similar manner but using genome-wide data, for those individuals that were also genotyped on the Affymetrix chip. These ancestry proportions were included as covariates in the SAC study group models.

Quality control of the Gambian data set revealed that missing genotypes were associated with having TB for a relatively large proportion of SNPs, which may be indicative of batch effects [65]. As this can be mitigated by the inclusion of principal components in statistical models, principal components were used to adjust the analysis, rather than ancestry proportions as was done for the SAC cohort. Principal components would adjust the models for both differences in ancestry and batch effects between cases and controls [66]. Principal component analysis of the Gambian study group showed associations between having TB and principal components 1, 2, 5, 6, 8, 9 and 10 (p-values < 0.05). These principal components were included as covariates in the Gambian study group models.

### Validation

Statistical modelling was used to identify gene pairs that most likely jointly modify the odds of having TB, and not to quantify the achieved level of statistical significance. Due to the large number of tests done in the SAC study group, and the limited size of the study group (especially the limited number of controls that were available for many of the tests), none of the interaction associations would be statistically significant if adjusted for multiple testing. In addition, many of the multiple testing methods that have been suggested in the literature have severe shortcomings. The straightforward Bonferroni adjustment is too stringent when several genetic associations are tested in the same study group due to correlation (LD) between markers [67, 68]. Alternative methods of correcting for multiple tests were also not feasible for this study. Firstly, roughly 2 million tests were done in differing subsets of individuals from the same study group, which complicates the use of multiple testing correction methods that do not rely on the simple adjustment of p-values by for example dividing by the number of tests done. Bayesian methods require a priori probability of association, which is not known. Due to the large number of tests that were done, permutation testing is also not feasible. Permutation testing is also inappropriate in the context of gene-gene interactions, as permutation based methods do not account for correlation between genotypes [69]. Furthermore, a large proportion of the tests were done on an unbalanced number of cases and controls, which may result in biased permutation-based calculation of p-values. A method to determine the number of effective independent tests when testing pairs of SNPs for interaction in a genome-wide context has also been proposed [55, 70]. This number of effective tests can then be used in a Bonferroni adjustment or to control the false discovery rate. The method does however not take into account that a gene may be tested in multiple gene-pair models, and the accuracy of the original method was evaluated using permutation testing, which may be inappropriate for interaction tests. Due to these reasons an appropriate alpha level was not determined, and we simply selected the top 20 unique gene pairs for validation in the Gambian study group. A similar strategy has been suggested by Kerr [71], albeit in the context of unbalanced microarray gene expression data. The selected models would be the most likely true positives, if any exist.

As patterns of linkage disequilibrium (LD) differ between populations, tag SNPs of causal variants may vary between the SAC and Gambian populations. A SNP associated in the SAC study group points to a region of LD, and any SNP within this region may be the causal SNP [72]. The 20 models that were selected for validation were therefore tested using all possible combinations of SNP pairs found in the region of the SNP tested in the SAC study group. Using a strategy similar to that of Shriner et al. [72] and Ramos et al. [73], SNPs used for validation of a SNP tested in the SAC study group was selected based on the following criteria: the SNPs were found in the same gene region, within 250 000 base pair positions of the SNP, and having a pairwise LD *r* ^2^ value of at least 0.3 with the SNP in SAC controls. Although some of the SNPs genotyped in candidate gene studies were selected for their putative functional effects, we note that all the variants in the top twenty models that were genotyped in candidate gene studies were originally selected as they were variants in a gene of interest, and not for their functional effects per se.

After selecting SNP pairs to test using this strategy, a resulting total of 245 regression models were fitted to the Gambian study group. P-values smaller than 0.05 were described as statistically significant.

### Software

Version information, web URLs and important parameter settings of the software packages used in this study are summarized in S3 Table.

PLINK was used for quality control of the SAC chip data set and Gambian chip data set [45]. The SAC and Gambian chip data sets were aligned to GRCh37 using a script and Affymetrix SNP information files available at http://www.well.ox.ac.uk/~wrayner/strand/.

ADMIXTURE was used to estimate ancestry proportions of the SAC study group [74]. For the Gambian study group, Eigenstrat was used to infer the top 10 principal components and test for association between these principal components and disease outcome [66]. Prior to estimating ancestry proportions and inferring principal components in the SAC chip data set and Gambian data set, PLINK was used to remove SNPs from the data set that were in LD, as this may lead to biased inference.

Biofilter was used to generate SNP pair combinations of genes that are known to interact based on experimental evidence, or that are found in the same biological pathway, ontological category or protein family. Only those combinations having three or more sources were used for testing interaction in the SAC chip data set. Biofilter was also used to find SNPs within gene regions that are available in the Gambian data set for validation of the top SAC gene-gene models.

The freely available R programming environment was used for statistical analyses, quality control of the SAC candidate genes and graphing [75]. The R *genetics* package was used to test for HWE in the SAC candidate genes and was also used to calculate pairwise LD *r* ^2^ and *D*′ values [76]. The R *haplo.stats* package was used to estimate allele combination frequencies in cases and controls [77]. The adjusted logits of the genotype combinations were estimated using the *effects* package [64]. Figures were created using the R *ggplot2* package [78].

## Results

The top 20 unique gene pair models discovered in the SAC cohort are summarized in Table 1. These models were identified using logistic regression, that tests whether the effect of a SNP on disease outcome is modified by the effect of another SNP, after taking into account (adjusting for) the main effects of the two SNPs. When encountering the same gene-gene model but with differing SNP pairs, only the gene-gene model with the smallest p-value is shown (4 models were excluded for this reason). SNP pairs and p-values of the corresponding highest scoring Gambian models are also reported in the table. As no suitable SNPs were available for some of the genes, some of the models could not be tested in the Gambian data set. Results of the top 250 SAC models and all 245 Gambian models are available in S4 Table. The effects of each of the SNPs in Table 1 were also tested individually in the relevant cohorts, and these single SNP association results are reported in S5 Table. Only two of the SNPs are individually associated with having TB in the SAC cohort (rs15842 and rs3740107 of models 7 and 20), but with a much lower level of significance than the interaction effect of the models (single SNP p-values of 1.49 × 10^−2^ and 1.64 × 10^−2^, interaction p-values of 6.23 × 10^−6^ and 1.37 × 10^−5^, respectively). Only one of the genes reported in Table 1, GRIK1, was identified by the top 36 single SNP associations from a previous genome-wide association study of the cohort [62]. By evaluating combinations of genes, a number of genes were identified that may play a role in TB pathogenesis, which would not have been evident if their effects were assessed individually.

**Table 1:** Top twenty interaction models. This table summarizes the top twenty interaction models identified in the SAC study group. P-values reflect the overall significance of the association between the genotype combinations and having TB, after adjusting for the main effects of the SNPs and covariates. A model of type C indicates a candidate gene pair, and a model of type B indicates a biofilter gene pair. These models were validated in the Gambian study group set using multiple SNPs found within the same gene regions, and the SNP pairs and p-values of the highest scoring Gambian models are reported. For some of the models, no SNPs were available in the Gambian data set for one or both of the genes (blank entries).

				**SAC**				**Gambian**			
	**Gene 1**	**Gene 2**	**Type**	**Nr cases**	**Nr controls**	**SNP 1**	**SNP 2**	**SNP 1**	**SNP 2**	**P-value**	**Nr tests**
**Model 1**	NRG1	NRG3	B	634	87	rs16879814	rs11191757	rs16879814	rs2224109	**0.0389**	1 × 12 = 12
**Model 2**	GRIK1	GRIK3	B	620	90	rs465555	rs3738085	rs460583	rs476894	**0.0476**	5 × 11 = 55
**Model 3**	SFTPD	NOD2	C	216	65	rs1923537	rs748855				
**Model 4**	IL23R	ATG4C	C	613	85	rs10489628	rs11208029	rs10489628	rs11208029	**0.0350**	1 × 3 = 3
**Model 5**	FUT8	B4GALT1	B	627	90	rs17102844	rs12342831	rs9323464	rs10758189	0.1399	4 × 7 = 28
**Model 6**	EXT1	EXT2	B	626	91	rs6469713	rs903509				
**Model 7**	ISG15	TLR8	C	271	321	rs15842	rs3761624				
**Model 8**	NCAM2	IRF8	C	620	87	rs8134735	rs8054065	rs8132838	rs147968	0.0794	4 × 2 = 8
**Model 9**	ANK1	ANK3	B	606	91	rs2102360	rs2393618				
**Model 10**	NELL1	NOS2	C	639	91	rs1377741	rs2297516	rs1377741	rs2314809	0.4098	1 × 2 = 2
**Model 11**	CADM3	SLC22A4	C	224	67	rs16841729	rs13179900				
**Model 12**	ANK2	ANK3	B	636	90	rs1354679	rs10821731	rs1354679	rs10761481	0.1544	5 × 18 = 90
**Model 13**	NELL1	CADM2	C	625	89	rs4614448	rs17024414	rs4614448	rs17024876	**0.0329**	3 × 7 = 21
**Model 14**	NLRC5	IL12RB1	C	231	245	rs289726	rs393548				
**Model 15**	PLCB1	PLCE1	B	633	91	rs708914	rs4918082	rs1703634	rs4918082	0.3165	2 × 1 = 2
**Model 16**	C1QA	TMEFF2	C	263	79	rs12033074	rs4077949				
**Model 17**	NELL1	CADM3	C	621	84	rs11025887	rs862991	rs12577018	rs862991	0.2107	2 × 1 = 2
**Model 18**	PDE2A	PDE4B	B	626	87	rs171021	rs536025	rs3781931	rs17423910	0.1169	2 × 4 = 8
**Model 19**	CHST11	CHSY3	B	623	87	rs17036205	rs32225	rs17036205	rs244745	**0.0401**	1 × 10 = 10
**Model 20**	SLC22A4	ALOX5	C	629	91	rs2306772	rs3740107	rs3792880	rs3780909	0.1117	4 × 1 = 4

**Fig 1:**
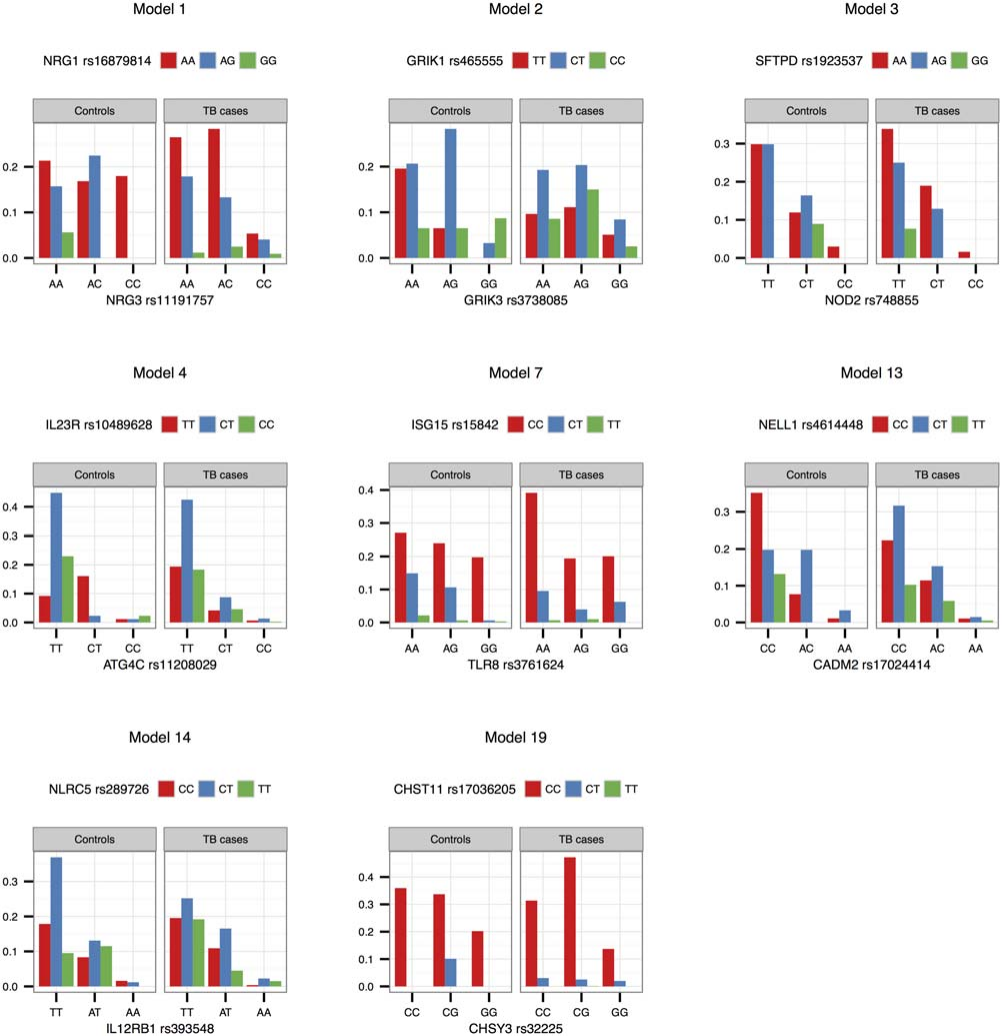
Genotype combination proportions in the SAC study group. The observed proportions of the nine possible SNP pair genotype combinations from models 1, 2, 3, 4, 7, 13, 14 and 19 are depicted in this figure, per cases and controls. Genotypes are ordered according to minor allele frequency, with the wildtype homozygote appearing first, and the rare homozygote appearing last.

**Fig 2:**
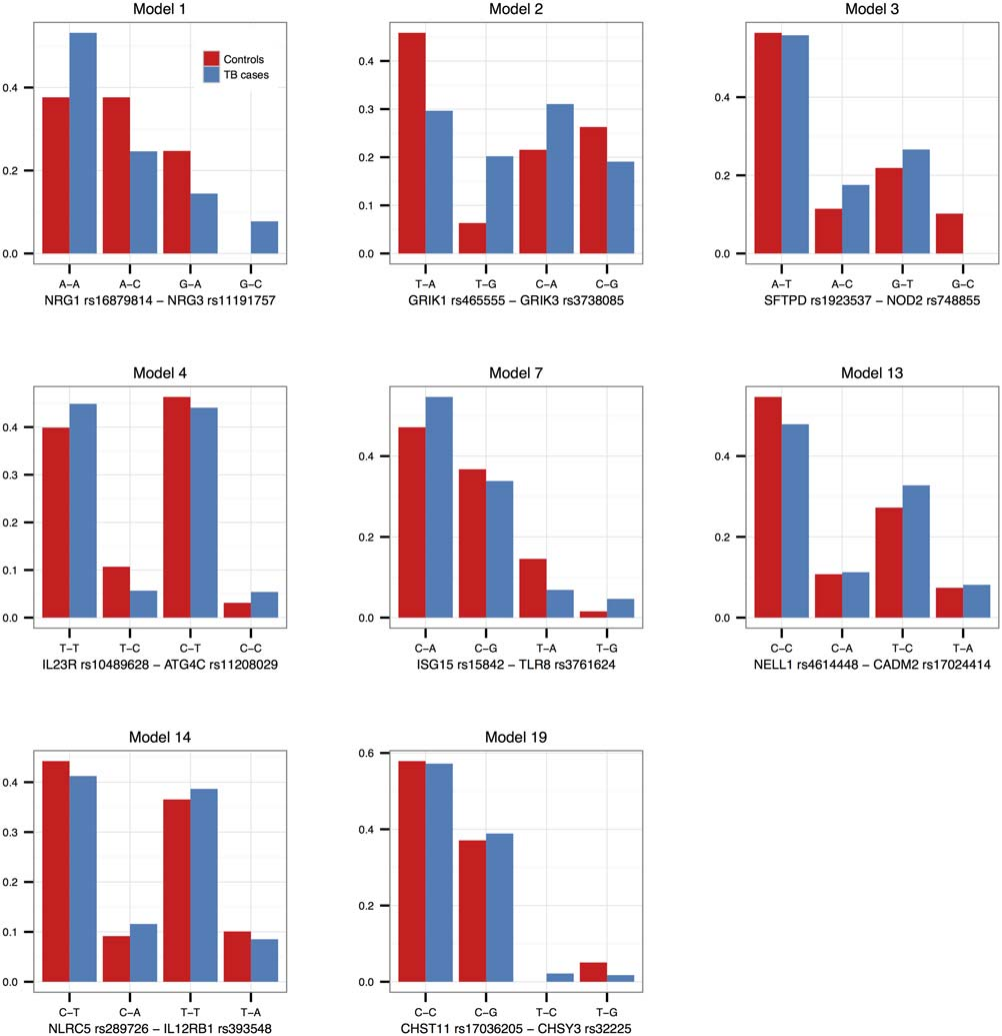
Allele combination frequencies in the SAC study group. The frequencies of the four possible SNP pair allele combinations from models 1, 2, 3, 4, 7, 13, 14 and 19 are depicted in this figure, per cases and controls. The frequencies were estimated using an EM-algorithm.

**Fig 3:**
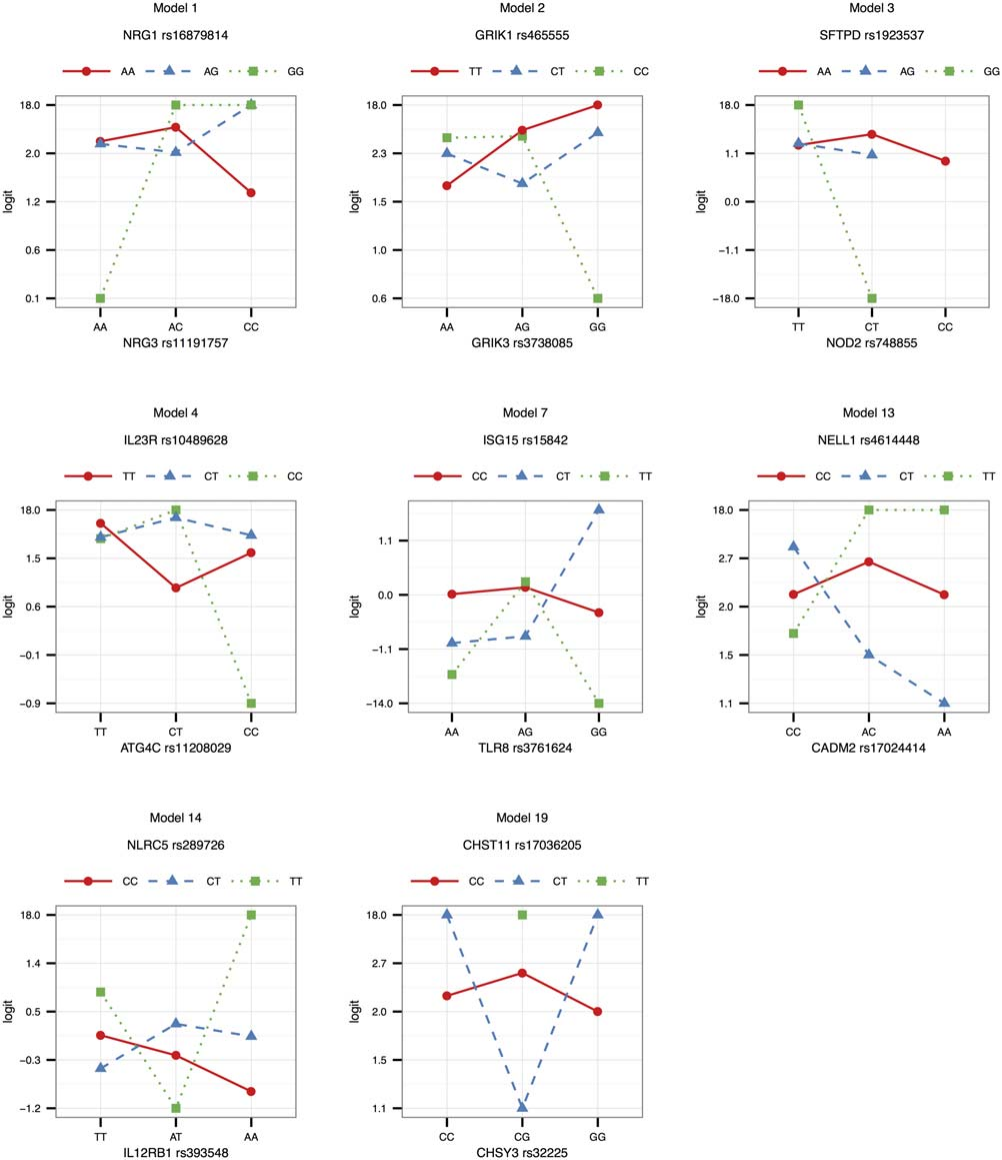
Effects in the SAC study group. The logits of genotype combinations from models 1, 2, 3, 4, 7, 13, 14 and 19 are depicted in this figure. Genotypes are ordered according to minor allele frequency, with the wildtype homozygote appearing first, and the rare homozygote appearing last. Non-parallel lines are indicative of interaction effects. The effects were estimated by absorbing the marginal effects of the SNPs into the SNP × SNP interaction term, and adjusting for the covariates included in the model by averaging over them.

**Fig 4:**
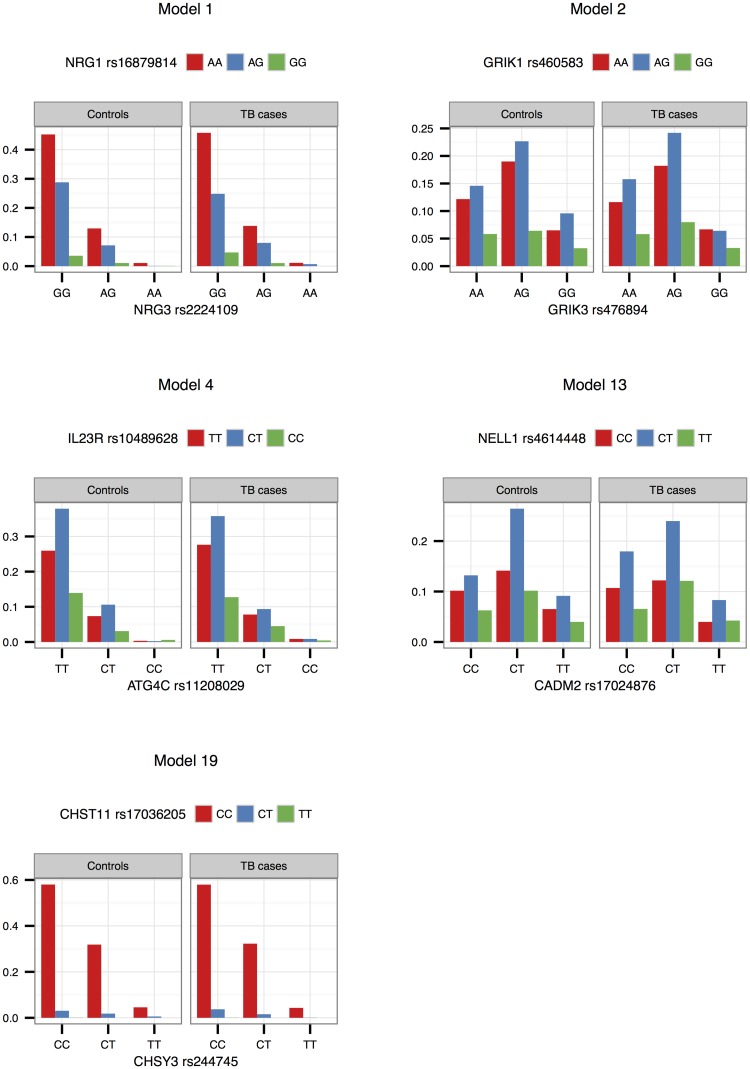
Genotype combination proportions in the Gambian study group. The observed proportions of the nine possible SNP pair genotype combinations from models 1, 2, 4, 13 and 19 are depicted in this figure, per cases and controls. Genotypes are ordered according to minor allele frequency, with the wildtype homozygote appearing first, and the rare homozygote appearing last.

**Fig 5:**
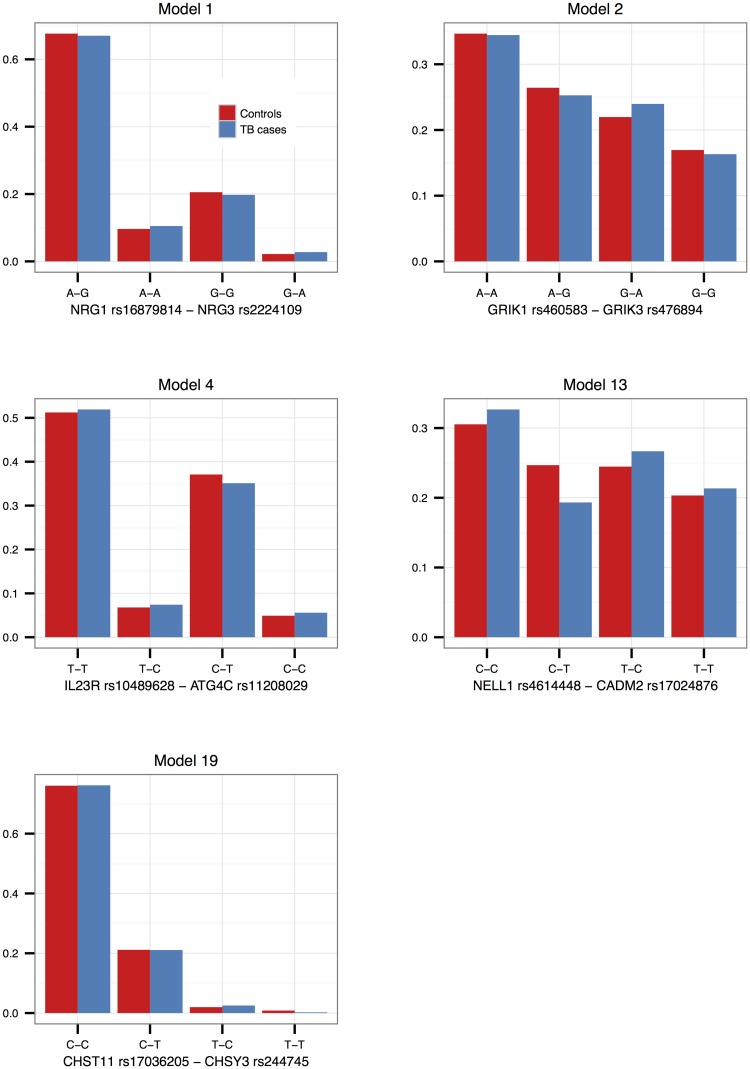
Allele combination frequencies in the Gambian study group. The frequencies of the four possible SNP pair allele combinations from models 1, 2, 4, 13 and 19 are depicted in this figure, per cases and controls. The frequencies were estimated using an EM-algorithm.

**Fig 6:**
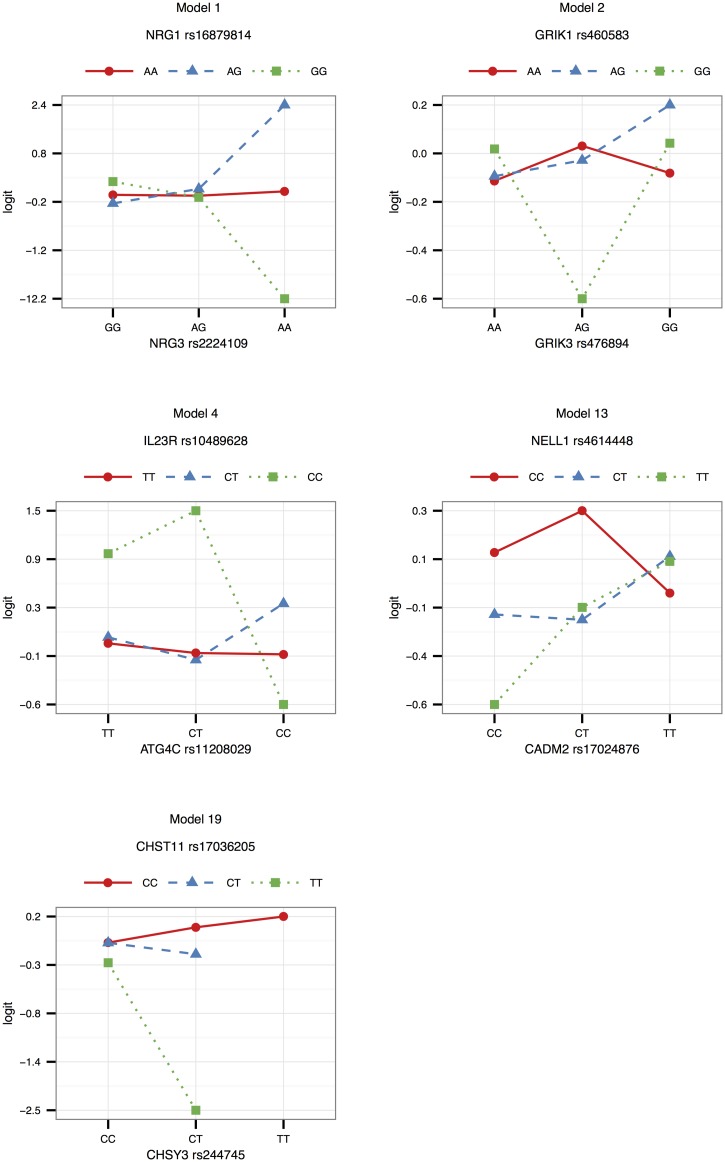
Effects in the Gambian study group. The logits of genotype combinations from models 1, 2, 4, 13 and 19 are depicted in this figure. Genotypes are ordered according to minor allele frequency, with the wildtype homozygote appearing first, and the rare homozygote appearing last. Non-parallel lines are indicative of interaction effects. The effects were estimated by absorbing the marginal effects of the SNPs into the SNP × SNP interaction term, and adjusting for the covariates included in the model by averaging over them.

Interaction effects observed in the SAC study group are illustrated in Figs 1–3 for validated models (p-value < 0.05 in the Gambian data set), as well as models that could not be validated due to lack of data, but that have interesting functional interpretations. Note that due to the differing SNP pairs used in the validation, as well as different allele frequencies and LD patterns in the two cohorts, the trend observed in a “validated” Gambian model may not necessarily reflect that of the corresponding SAC model, and we use the term here to imply that there is evidence in both cohorts that the gene pair jointly modifies the odds of having TB. Figs 1 and 2 show the frequencies of the genotype and allele combinations in cases and controls. As per the definition of interaction, the allele combination graphs demonstrate the reversal of effects in cases and controls, e.g. if the SNP 1 allele 1—SNP 2 allele 1 combination has a lower frequency in controls compared to cases, then the SNP 1 allele 1—SNP 2 allele 2 combination has a higher frequency in controls compared to cases, i.e. the effect of allele 1 of SNP 1 is modified by the SNP 2 allele. Fig 3 depicts the joint effect that genotype combinations have on the odds of having TB, after adjustment for covariates; non-parallel lines being indicative of interaction effects. For example, model 7 in Fig 3 shows that compared to the CT-AG genotype combination, the CT-GG combination increases the odds of having TB, whereas compared to the TT-AG combination, the TT-GG combination decreases the odds of having TB. Put another way, depending on whether the first SNP has one or two copies of the rare allele T, the effect of having two instead of one copies of the rare allele G for that SNP may increase or decrease the odds of having disease. The frequencies and effects in the SAC study group for the remaining top models are depicted similarly in supplementary figures (S1–S3 Figs), and Figs 4–6 show the frequencies and effects of the validated Gambian models. Below we highlight models that were validated in the Gambian data set as well as three models that could not be tested, but that have interesting functional effects.

The *NRG1—NRG3* (Neuregulin 1 and 3) interaction effect observed in the SAC study group (model 1, p-value 8.32 × 10^−7^) was also detected in the Gambian study group (p-value 0.0389). The SAC and WTCCC *NRG1* SNP is the same, and the Gambian *NRG3* SNP is located 66 235 base pairs upstream from the SAC *NRG3* SNP. In both the SAC and Gambian study groups, compared to the GG-AC/GG-AG combination, the GG-AA combination decreases the odds of having TB and the AG-AA combination increases the odds of having TB. The same pattern is thus observed, albeit using a different SNP in the second gene in the Gambian study group. Studies investigating the link between NRG1 and schizophrenia have demonstrated that NRG1 has a functional effect on the immune system by influencing immune cell adhesion [79] and the concentration of autoantibodies and pro-inflammatory cytokines in plasma [80]. Gene-gene interaction between *NRG1* and *NRG3* has also been observed in a schizophrenia study [81], and according to the NCBI BioSystems database, NRG3 may also be involved in the immune system.

An interaction between the *GRIK1* and *GRIK3* (glutamate receptor 1 and 2) genes was also detected in both study groups (model 2, SAC p-value 1.62 × 10^−6^ and Gambian p-value 0.0476). The TT-GG genotype combination was observed only in cases (5%) and the T-G allelic combination is more frequent in cases compared to controls (21% vs. 7%). The Gambian *GRIK1* SNP is located 3 478 base pairs downstream from the SAC *GRIK1* SNP and the Gambian *GRIK3* SNP is located 1 286 base pairs downstream from the SAC *GRIK3* SNP. *GRIK1* has been associated with susceptibility to diabetes [82], and according to T1DBase (a database focused on the genetics and genomics of type 1 diabetes susceptibility, http://www.t1dbase.org), *GRIK3* is also a putative diabetes susceptibility gene. Having diabetes increases susceptibility to TB [83], and this may explain the *GRIK1—GRIK3* interaction association we observed in the data.

Another model that was observed in both study groups is the interaction between *IL23R* (interleukin-23 receptor) and *ATG4C* (autophagy related 4C, cysteine peptidase) (model 4, SAC p-value 2.18 × 10^−6^ and Gambian p-value 0.0350). T helper 17 (Th17) cells are subsets of activated CD4+ (cluster of differentiation 4 plus) T cells that mediates the recruitment of macrophages to infected tissues. The Th17 response to *M. tuberculosis* infection is largely dependent on interleukin-23 [84]. ATG4C is thought to play a role in autophagy [85] and is up-regulated when TRPV1 (transient receptor potential cation channel subfamily V member 1) channels are expressed on CD4+ T cells [86, 87]. The *IL23R* and *ATG4C* gene products may therefore both be involved in the Th17 response to *M. tuberculosis*. The same SNPs are used in both models, with the CC-CC genotype combination decreasing the odds of having TB in both cohorts, compared to the CC-CT and CC-TT combinations. Both of the SNPs are located on chromosome 1p31 and are 6 centimorgans (4 451 385 base pairs) apart. Linkage disequilibrium between the SNPs is high in SAC controls (*D*′ = 0.5451) but not in SAC cases (*D*′ = 0.0136), and low in both Gambian controls and cases (*D*′ = 0.0011 and *D*′ = 0.0386 respectively).

Interaction between the *NELL1* (neural epidermal growth factor-like 1) and *CADM2* (cell adhesion molecule 2) genes is also evident in both the SAC and WTCCC study groups (model 13, SAC p-value 1.14 × 10^−5^ and Gambian p-value 0.0329), as well as interaction between the *NELL1* and *CADM3* (cell adhesion molecule 3) genes, although the latter was not validated in the Gambian study group (model 17, SAC p-value 1.26 × 10^−5^). The same trend between the effects of the heterozygote genotype combinations is observed in both study groups for model 13, compared to the pairing with the wildtype homozygote genotype (CC) of the second SNP in the pair. A large degree of homology exists between the *CADM1*, *CADM2* and *CADM3* genes [88, 89] and CADM1 has been shown to affect the expression of interleukin-22 [90]. *NELL1* is expressed in pre-B cell development [91] and has been associated with inflammatory bowl disease, a complex auto-immune disorder [92]. The link between the interplay of these genes and TB susceptibility is however not clear. We also note that in the Gambian cohort, the NELL1 single SNP association signal is stronger than the interaction effect (single SNP p-value of 0.0022, see S5 Table, vs. interaction p-value of 0.0329).

The *CHST11*—*CHSY3* (carbohydrate (chondroitin 4) sulfotransferase 11—chondroitin sulfate synthase 3) gene pair interaction was also detected in both study groups (model 19, SAC p-value 1.34 × 10^−5^ and Gambian p-value 0.0401). Uhlin et al. [93] showed that expression of *CSPG* (chondroitin sulfate proteoglycan) decreased when monocyte-derived macrophages are treated with interferon-gamma. CSPG is composed of a protein core and a chondroitin sulfate side chain. According to the NCBI BioSystems database, both the *CHST11* and *CHSY3* genes are involved in the chondroitin sulfate pathway. The CT-CG genotype combination has a higher frequency in SAC controls compared to cases (10% vs. 3%). The *CHST11* SNP is the same in the SAC and Gambian models, and the *CHSY3* Gambian SNP is 8 683 base pairs upstream from the SAC *CHSY3* SNP. The CT-TT combination was observed in 7 Gambian controls and in 1 case.

NF-*κ*B (nuclear factor kappa-light-chain-enhancer of activated B cells) signalling plays in an important role in the host defense against *M. tuberculosis* infection [94]. Both the *SFTPD* (surfactant protein D) and *NOD2* (nucleotide-binding oligomerization domain containing 2) genes are involved in this pathway [95, 96]. Interaction between these genes was identified in the SAC study group (model 3), but no suitable SNPs were available for validation in the Gambian data set. The GG-CT genotype combination is present only in SAC controls (9%), whereas the GG-TT genotype combination is present only in SAC cases (8%).

The *ISG15* (ISG15 ubiquitin-like modifier) and *TLR8* (Toll-like receptor 8) gene products may also affect NF-*κ*B signalling. ISG15 stimulates interferon-gamma production [97] which in turn activates NF-*κ*B signalling [98]. It has also been postulated that TLR8 activates NF-*κ*B signalling [99]. This gene pair showed interaction in the SAC study group (model 7) but could not be validated in the Gambian study group due to lack of suitable SNPs. The CT-GG genotype combination occurs in 7% of SAC cases, but in only 1% of controls.

Another model of interest that could not be validated in the Gambian cohort due to lack of suitable SNPs is the interaction between *NLRC5* (NLR family, CARD domain containing 5) and *IL12RB1* (interleukin 12 receptor, beta 1 genes) (model 14). The interferon-gamma/interleukin-12 pathway is an important component of the immune defense against mycobacterial infections [100]. IL12RB1 is a receptor of interleukin-12, and it has been shown that the *NLRC5* promoter region is responsive to interferon-gamma, which implies that NLRC5 may function as a molecular switch of interferon-gamma activation [101]. The TT-AT genotype combination has a higher frequency in SAC controls compared to cases (12% vs. 5%), whereas the TT-TT combination has a higher frequency in SAC cases compared to controls (19% vs. 10%).

Finally, we explored whether allelic encoding of the SNPs may better explain the interactions detected in the SAC cohort. S6 Table summarizes the p-values of the four degrees of freedom genotypic tests for interaction that was used to select the top 20 models, as well as the p-values of the corresponding allelic models that attained the highest level of significance. It is evident from these results that dominant/recessive effects may in some cases better encapsulate the interaction effects observed in the data, and this is depicted in S4 Fig. We note that for all of the five models that were successfully validated in the Gambian cohort, the genotypic test for interaction achieved the highest level of significance.

## Discussion

The South African Coloured population is an ideal cohort for the discovery of TB susceptibility genetic variants, since they received genetic contributions from diverse source populations that may differ in their susceptibility to TB. Seldin et al. [102] has argued that it is important to study the role of complex disease epistasis in such admixed populations, and that this may well uncover novel interactions that are not detectable in the source populations. In this study we used SAC genome-wide data as well as genotypes from a large number of candidate gene studies to discover genetic variants that may jointly modify the odds of having TB. We limited our search space to biologically plausible gene pair models and used statistical modelling to detect interactions, allowing us to adjust for known differences between cases and controls (age, gender and ancestry). Our study does however have a number of limitations, which we discuss below.

Genotypes available for testing the gene pair models were limited to SNPs that were genotyped on the Affymetrix 500K SNP chip as well as candidate gene studies performed in our group. The Affymetrix chip was originally designed based on LD patterns in European populations, and as a result the proportion of variants that are tagged in African populations may be much reduced [103].

Minor allele frequencies of the SNPs representing genes in the top models are in general quite different between the SAC and Gambian cohorts (S5 Table). Of the five models that were successfully validated, only one of the models was validated using exactly the same SNPs, three models were validated with one SNP in common, and one model was validated with completely different SNPs. Patterns of LD are also likely to differ between the SAC and Gambian cohorts, and according to NCBI, none of the SNPs in the top result set have functional effects, implying that the SNPs may all be tagging causative variants. Due to these factors, it is difficult to compare the effect sizes between the two cohorts directly. Indeed, two studies of the association between rs1024611 and TB susceptibility found that the association was statistically significant, but that the G allele of the SNP was protective in the one population, and increased susceptibility in the other population. The true causal variant that rs1024611 was in LD with was later identified, which may explain the opposite effects observed in the two populations [4]. The complexity of disentangling such different effects would be exacerbated in the context of interaction modelling. This could be alleviated to some degree if a higher density of markers was available, which would better capture causative variants. A denser marker panel could be imputed, but in our opinion, this exercise would likely be error-prone. Additional uncertainty would be introduced through imputation, and the proportion of genotype inaccuracies could potentially be large. Imputation relies on linkage disequilibrium between markers, which may not be captured accurately by the Affymetrix 500K SNP for our study cohorts, as a result of the chip’s European-centric design. In addition, the San has contributed a large amount of genetic material to the SAC [61, 104–108], and due to the lack of large high density reference panels for the San, this may contribute to additional inaccuracies in imputation of the SAC data set.

It is difficult to quantify the precise levels of significance of our results, due to the large number of tests in the SAC data set, and the limitations of methods available to correct for multiple testing. If we were to use a multiple testing correction similar to the one used by Emily et al. [55], despite its limitations, we would have to show that the number of effective independent tests was 60 000, for the topmost model to be significant at a Bonferroni adjusted alpha level of 0.05 (0.05/60000 = 8.33 × 10^−7^, p-value of model 1 was 8.33 × 10^−7^). Given that roughly 2 million models were tested, and that Emily et al. found that the number of effective independent tests was approximately six times less than the actual number of tests, it is unlikely that we would be able to demonstrate this (a 33 times reduction would be required). The SAC genome-wide data set was originally genotyped with a view to perform a case-only admixture mapping study, and for this reason, a limited number of controls was available for many of the two SNP interaction tests. Whilst the available group sizes are sufficient to detect two-SNP interactions, it is unlikely that any of the results would achieve statistical significance. For validation models fitted to the Gambian data set, we describe tests with p-values < 0.05 as statistically significant. As 20 gene pair tests were done (ranging between 2 to 90 SNP pair tests per gene pair, see Table 1), none of these results would survive correction for multiple testing, and we note that the results should be interpreted with caution. We do however argue that both the SAC and Gambian data sets do not merely constitute random data, and that our results may contain actual associations that should not be dismissed [17]. Given the complex nature of the immune system defence against TB and the role that gene-gene interactions might play in this, it is plausible that some of our top results represent real biological phenomena worthy of further investigation.

Seven of the top models identified in the SAC study group could not be tested in the Gambian study group due to the absence of suitable SNPs. Eight of the models were not successfully validated. These results could be false positives, but their validation failure could also be ascribed to a number of other reasons. Differing patterns of LD between the SAC and Gambian populations and lack of SNP coverage by the Affymetrix 500K SNP chip, which our SNP selection strategy could not fully compensate for, could result in unsuccessful validation. The *M. tuberculosis* genome varies substantially across geographic regions [109], including between South and West Africa, and it has been hypothesised that interactions between host and pathogen differ between population groups [8, 109, 110]. Due to the heterogeneity of the source populations that contributed to the formation of the SAC, it is also possible that some interactions involved in TB susceptibility are unique to the SAC [102]. In spite of the limitations discussed above, five of the top twenty models were indeed validated in an independent Gambian case-control data set, although the levels of significance of the validation models were not very small (p-value < 0.05 but > 0.01). These models indicate that TB susceptibility is modified by interplay between the *NRG1—NRG3*, *GRIK1—GRIK3* and *IL23R—ATG4C* gene pairs, and the fact that the validation population is ethnically very different could imply that the interactions found have universal relevance.

The frequencies and effects are depicted graphically to aid interpretation, but as the SNPs used in the models are tag variants that may not have causative functional effects, the biological implications of the models are not yet fully understood. Validation in other populations, fine-mapping of the causal variants and functional studies will be required to elucidate our findings.

## Conclusion

In this study we investigated the role of gene-gene interactions in TB susceptibility in the South African Coloured population. To our knowledge, in terms of number of genetic loci considered, this is the largest study of gene-gene interactions and TB susceptibility that has been reported to date. We report a number of interesting results, five of which were validated in an independent cohort from the Gambia.

## Supporting Information

S1 Fig Genotype combination proportions in the SAC study group. The observed proportions of the nine possible SNP pair genotype combinations from models 5, 6, 8, 9, 10, 11, 12, 15, 16, 17, 18 and 20 are depicted in this figure, per cases and controls. Genotypes are ordered according to minor allele frequency, with the wildtype homozygote appearing first, and the rare homozygote appearing last.
(PDF)

S2 Fig Allele combination frequencies in the SAC study group. The frequencies of the four possible SNP pair allele combinations from models 5, 6, 8, 9, 10, 11, 12, 15, 16, 17, 18 and 20 are depicted in this figure, per cases and controls. The frequencies were estimated using an EM-algorithm.
(PDF)

S3 Fig Effects in the SAC study group. The logits of genotype combinations from models 5, 6, 8, 9, 10, 11, 12, 15, 16, 17, 18 and 20 are depicted in this figure. Genotypes are ordered according to minor allele frequency, with the wildtype homozygote appearing first, and the rare homozygote appearing last. Non-parallel lines are indicative of interaction effects. The effects were estimated by absorbing the marginal effects of the SNPs into the SNP × SNP interaction term, and adjusting for the covariates included in the model by averaging over them.
(PDF)

S4 Fig Dominant/recessive combination proportions in the SAC study group. The observed proportions of SNP pair genotype combinations from models 3, 5, 7, 8, 9, 16, 17 and 18 are depicted in this figure, per cases and controls. Recessive/dominant effects in these models may better explain the interactions observed in the cohort (smaller p-values were achieved compared to the genotypic models, and the best models with 1 or more recessive or dominant encodings listed in S5 Table are presented in this figure). Rare homozygotes and heterozygotes are combined to represent dominant encoding of alleles, and wild type homozygotes and heterozygotes are combined to represent recessive encoding of alleles. For dominant and recessive allelic encodings of SNPs, the last genotype presented therefore reflects an encoding of 1.
(PDF)

S1 Table TB susceptibility candidate gene association studies. The table summarizes the total number of samples that were successfully genotyped in each candidate gene study and how many samples have complete confounder information (age, gender and ancestry).
(PDF)

S2 Table Age and gender in the tuberculosis study groups. P-values were calculated using logistic regression.
(PDF)

S3 Table Software used in this study. A summary listing web URLs, version information and important parameter settings of software used in this study.
(PDF)

S4 Table Results of the top 250 SAC models and all 245 Gambian models. A spreadsheet with two worksheets, showing the results of the top 250 SAC models and the 245 Gambian models that were used for validation.
(XLS)

S5 Table Single SNP summary of the top model SNPs in the SAC and Gambian cohorts. This table provides a summary of each SNP’s individual minor allele frequency (MAF) and association with having TB.
(PDF)

S6 Table P-values of the top models in the SAC cohort. The genotypic model p-values, which were used to select the top 20 models, are presented in this table. The p-values of the corresponding allelic interaction models that achieved the smallest p-values are also shown.
(PDF)
