# Supplementary material for: Investigating the Role of Gene-Gene Interactions in TB Susceptibility
Source: PLoS One. 2015 Apr 28;10(4):e0123970. doi: 10.1371/journal.pone.0123970 (PMC4412713; doi:10.1371/journal.pone.0123970)
Supplement: S1 Fig — The observed proportions of the nine possible SNP pair genotype combinations from models 5, 6, 8, 9, 10, 11, 12, 15, 16, 17, 18 and 20 are depicted in this figure, per cases and controls. Genotypes are ordered according to minor allele frequency, with the wildtype homozygote appearing first, and the rare homozygote appearing last. (PDF) [file pone.0123970.s001.pdf]

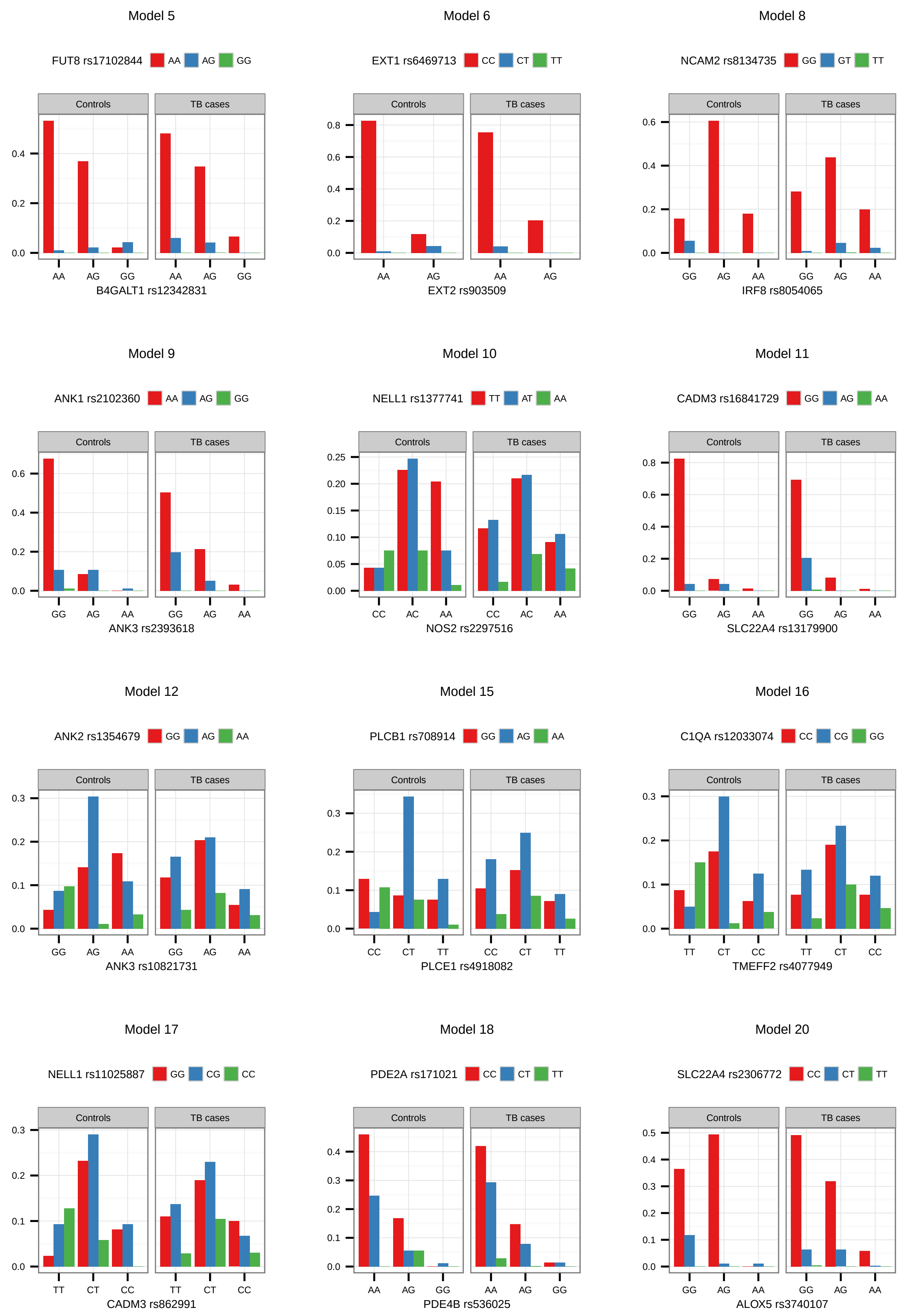
Model 5
FUT8 rs17102844 AA AG GG
Controls
TB cases
0.0 0.2 0.4
AA AG GG AA AG GG
B4GALT1 rs12342831
Model 6
EXT1 rs6469713 CC CT TT
Controls
TB cases
0.0 0.2 0.4 0.6 0.8
AA AG AA AG
EXT2 rs903509
Model 8
NCAM2 rs8134735 GG GT TT
Controls
TB cases
0.0 0.2 0.4 0.6
GG AG AA GG AG AA
IRF8 rs8054065
Model 9
ANK1 rs2102360 AA AG GG
Controls
TB cases
0.0 0.2 0.4 0.6
GG AG AA GG AG AA
ANK3 rs2393618
Model 10
NELL1 rs1377741 TT AT AA
Controls
TB cases
0.00 0.05 0.10 0.15 0.20 0.25
CC AC AA CC AC AA
NOS2 rs2297516
Model 11
CADM3 rs16841729 GG AG AA
Controls
TB cases
0.0 0.2 0.4 0.6 0.8
GG AG AA GG AG AA
SLC22A4 rs13179900
Model 12
ANK2 rs1354679 GG AG AA
Controls
TB cases
0.0 0.1 0.2 0.3
GG AG AA GG AG AA
ANK3 rs10821731
Model 15
PLCB1 rs708914 GG AG AA
Controls
TB cases
0.0 0.1 0.2 0.3
CC CT TT CC CT TT
PLCE1 rs4918082
Model 16
C1QA rs12033074 CC CG GG
Controls
TB cases
0.0 0.1 0.2 0.3
TT CT CC TT CT CC
TMEFF2 rs4077949
Model 17
NELL1 rs11025887 GG CG CC
Controls
TB cases
0.0 0.1 0.2 0.3
TT CT CC TT CT CC
CADM3 rs862991
Model 18
PDE2A rs171021 CC CT TT
Controls
TB cases
0.0 0.1 0.2 0.3 0.4
AA AG GG AA AG GG
PDE4B rs536025
Model 20
SLC22A4 rs2306772 CC CT TT
Controls
TB cases
0.0 0.1 0.2 0.3 0.4 0.5
GG AG AA GG AG AA
ALOX5 rs3740107
