# Supplementary figures and images for: Investigating the Role of Gene-Gene Interactions in TB Susceptibility
Source: PLoS One. 2015 Apr 28;10(4):e0123970. doi: 10.1371/journal.pone.0123970 (PMC4412713; doi:10.1371/journal.pone.0123970)

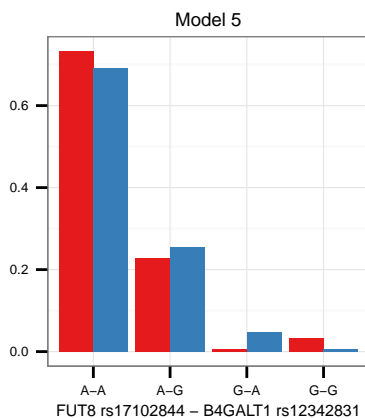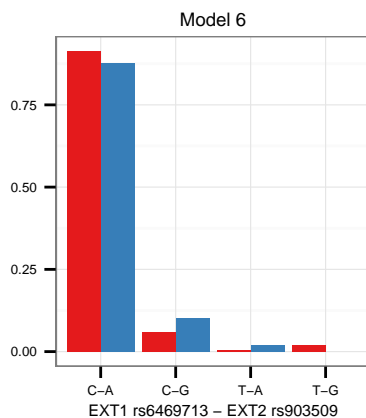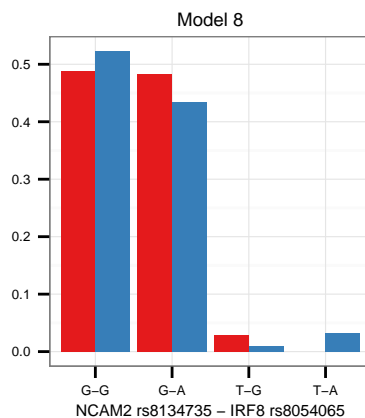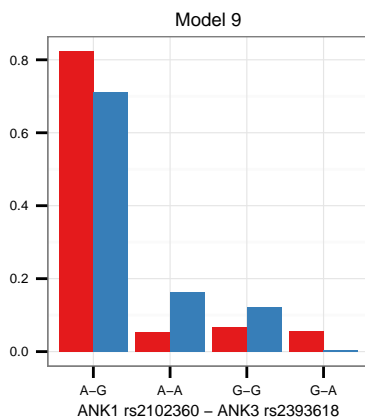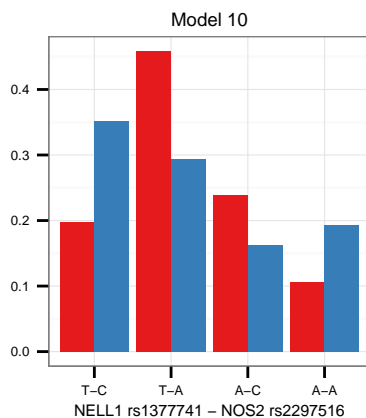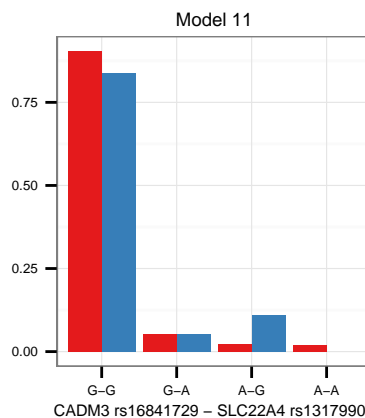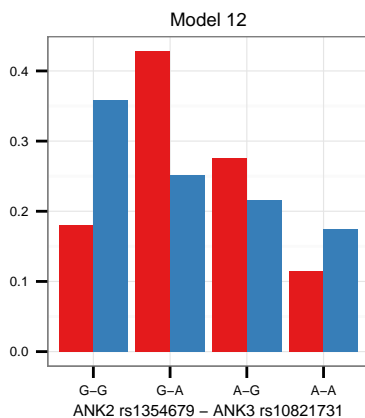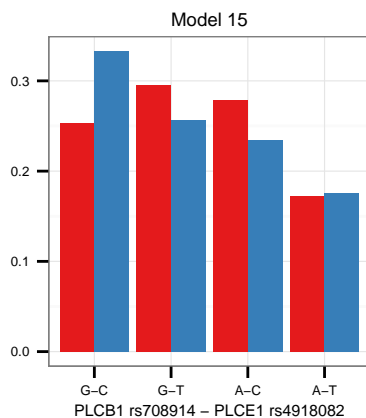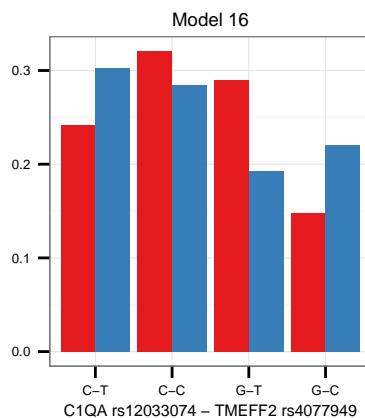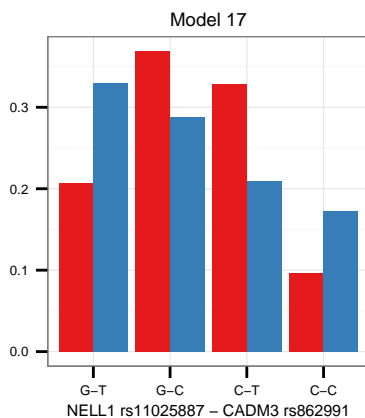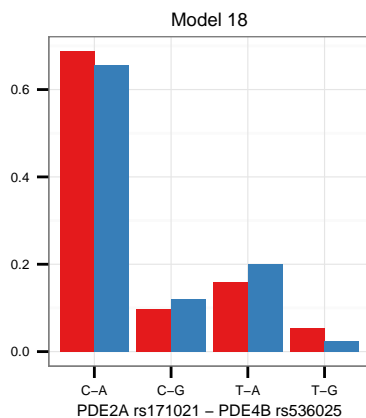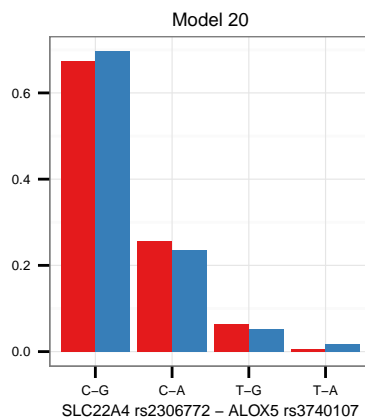

Supplement: S2 Fig — The frequencies of the four possible SNP pair allele combinations from models 5, 6, 8, 9, 10, 11, 12, 15, 16, 17, 18 and 20 are depicted in this figure, per cases and controls. The frequencies were estimated using an EM-algorithm. (PDF) [file pone.0123970.s002.pdf]

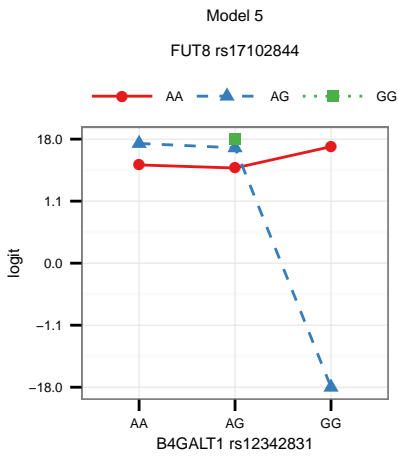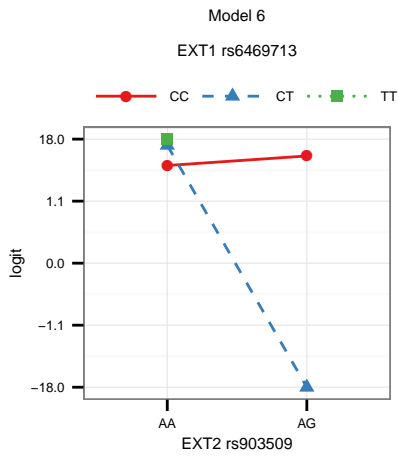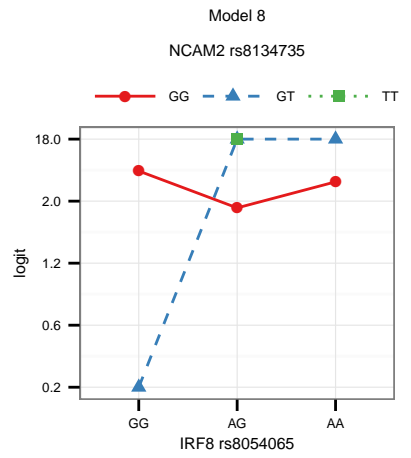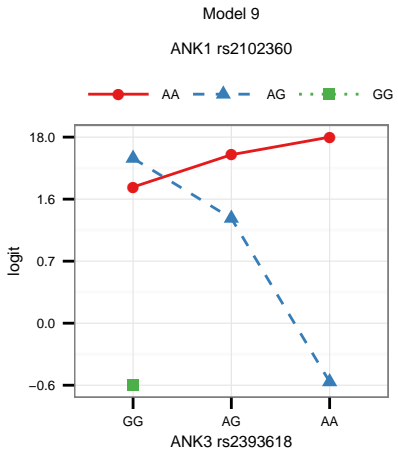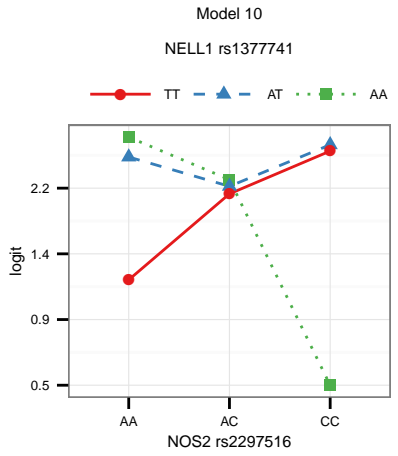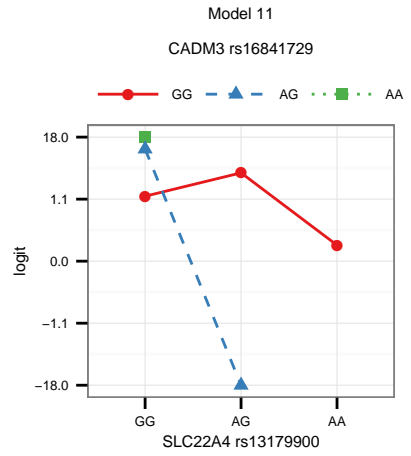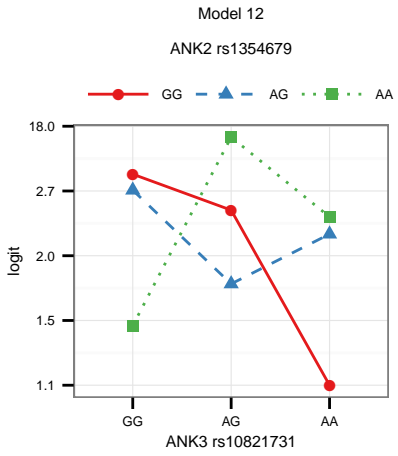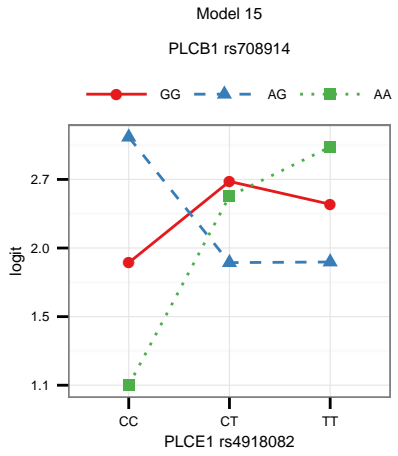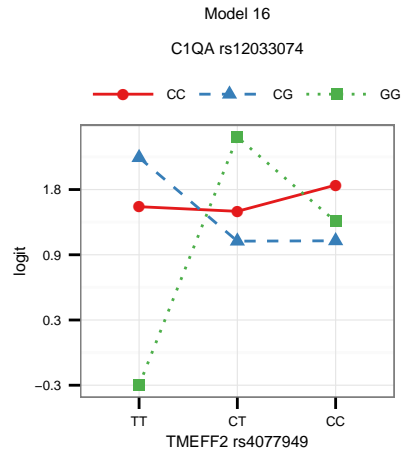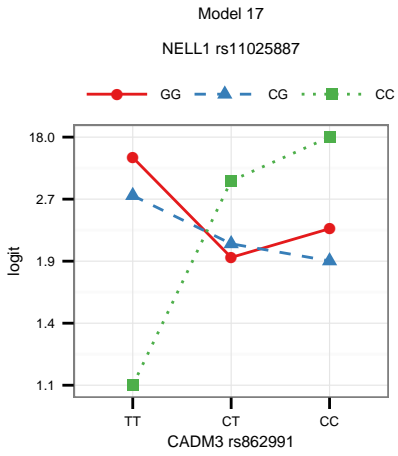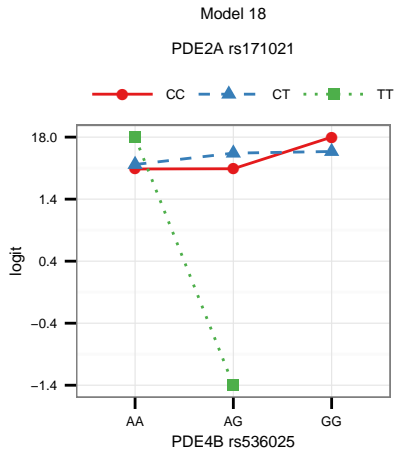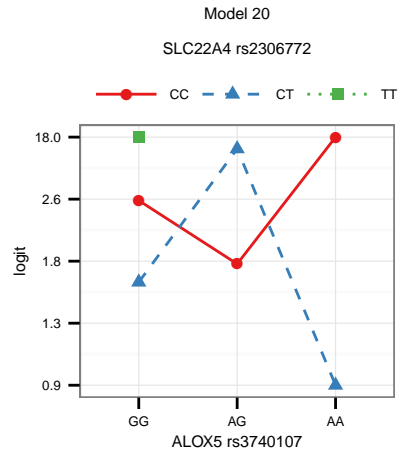

Supplement: S3 Fig — The logits of genotype combinations from models 5, 6, 8, 9, 10, 11, 12, 15, 16, 17, 18 and 20 are depicted in this figure. Genotypes are ordered according to minor allele frequency, with the wildtype homozygote appearing first, and the rare homozygote appearing last. Non-parallel lines are indicative of interaction effects. The effects were estimated by absorbing the marginal effects of the SNPs into the SNP × SNP interaction term, and adjusting for the covariates included in the model by averaging over them. (PDF) [file pone.0123970.s003.pdf]
