# Supplementary material for: Investigating the Role of Gene-Gene Interactions in TB Susceptibility
Source: PLoS One. 2015 Apr 28;10(4):e0123970. doi: 10.1371/journal.pone.0123970 (PMC4412713; doi:10.1371/journal.pone.0123970)
Supplement: S4 Fig — The observed proportions of SNP pair genotype combinations from models 3, 5, 7, 8, 9, 16, 17 and 18 are depicted in this figure, per cases and controls. Recessive/dominant effects in these models may better explain the interactions observed in the cohort (smaller p-values were achieved compared to the genotypic models, and the best models with 1 or more recessive or dominant encodings listed in S5 Table are presented in this figure). Rare homozygotes and heterozygotes are combined to represent dominant encoding of alleles, and wild type homozygotes and heterozygotes are combined to represent recessive encoding of alleles. For dominant and recessive allelic encodings of SNPs, the last genotype presented therefore reflects an encoding of 1. (PDF) [file pone.0123970.s004.pdf]

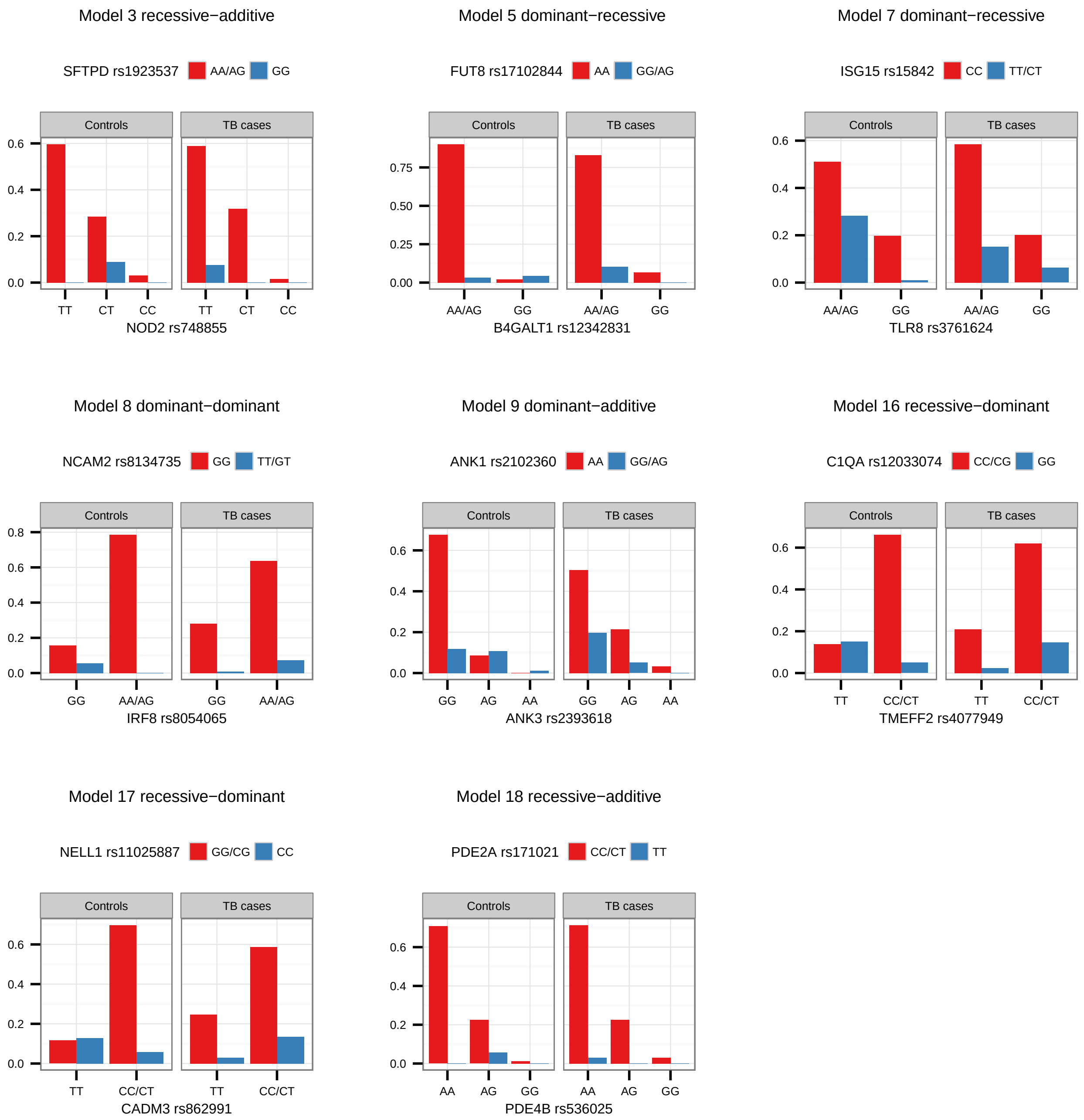
Model 3 recessive−additive
SFTPD rs1923537 AA/AG GG
Controls
TB cases
0.0 0.2 0.4 0.6
TT CT CC TT CT CC
NOD2 rs748855
Model 5 dominant−recessive
FUT8 rs17102844 AA GG/AG
Controls
TB cases
0.00 0.25 0.50 0.75
AA/AG GG AA/AG GG
B4GALT1 rs12342831
Model 7 dominant−recessive
ISG15 rs15842 CC TT/CT
Controls
TB cases
0.0 0.2 0.4 0.6
AA/AG GG AA/AG GG
TLR8 rs3761624
Model 8 dominant−dominant
NCAM2 rs8134735 GG TT/GT
Controls
TB cases
0.0 0.2 0.4 0.6 0.8
GG AA/AG GG AA/AG
IRF8 rs8054065
Model 9 dominant−additive
ANK1 rs2102360 AA GG/AG
Controls
TB cases
0.0 0.2 0.4 0.6
GG AG AA GG AG AA
ANK3 rs2393618
Model 16 recessive−dominant
C1QA rs12033074 CC/CG GG
Controls
TB cases
0.0 0.2 0.4 0.6
TT CC/CT TT CC/CT
TMEFF2 rs4077949
Model 17 recessive−dominant
NELL1 rs11025887 GG/CG CC
Controls
TB cases
0.0 0.2 0.4 0.6
TT CC/CT TT CC/CT
CADM3 rs862991
Model 18 recessive−additive
PDE2A rs171021 CC/CT TT
Controls
TB cases
0.0 0.2 0.4 0.6
AA AG GG AA AG GG
PDE4B rs536025
