# Supplementary material for: Investigating the Role of Gene-Gene Interactions in TB Susceptibility
Source: PLoS One. 2015 Apr 28;10(4):e0123970. doi: 10.1371/journal.pone.0123970 (PMC4412713; doi:10.1371/journal.pone.0123970)
Supplement: S1 Table — The table summarizes the total number of samples that were successfully genotyped in each candidate gene study and how many samples have complete confounder information (age, gender and ancestry). (PDF) [file pone.0123970.s005.pdf]

			TB cases			Controls
**Study**	**Year**	**Genes**	**Total**	**Complete**	**Total**	**Complete**
Rossouw et al. [1]	2003	IFNG	393	302	286	231
Hoal et al. [2]	2004	SLC11A1, SLC11A2	429	324	436	288
Babb et al. [3]	2007	SP110	379	334	304	268
Barreiro et al. [4]	2007	CD209	339	286	227	202
Möller, unpublished [5], Möller et al. [6, 7, 8, 9, 10, 11]	2007-2011	ATG16L1, BTNL2, NOD2, CCL2, CTLA4, CTSZ, FCRL3, FZD5, IL10, IL12B, IL12RB1, IL12RB2, IL18, IL1RN, IL23R, IL4, IL6ST, INSIG2, ABCB1, CIITA, MS4A2, NELL1, NOS2, P ADI4, PPARG, PTGER4, PTPN22, RUNX1, SH2D1A, SLC22A4, SLC22A5, SOCS3, TEX264, TLR2, TLR4, TNF, TNFRSF1A, TNFRSF1B, TNFSF15, WNT5A	781	584	703	390
Adams et al. [11]	2011	MC3R	439	386	505	410
Babb, unpublished [12]	2007	CCR5, CCL5, CXCL12	312	229	228	178
De Wit, unpublished [13]	2009	IFNGR1, IL8, RANTES	397	303	289	231
Salie, unpublished [14]	2010	ANXA11, CADM1, CADM2, CADM3, NCAM2	382	341	398	344
Lucas, unpublished [15]	2011	TLR8, TLR9	479	439	496	447
Wagman, unpublished [16]	2011	MARCO, SFTPD	383	343	395	343
Bruiners, unpublished [17]	2012	C1QA, C1QB	472	433	477	431
Unpublished	2001	SPP1	206	148	124	64
Unpublished	2002	SFTPD	161	124	144	75
Unpublished	2003	IL8	214	156	149	112
Unpublished	2003	IFNGR1, IFNGR2	373	275	348	226
Unpublished	2004	CO2REGION, HS3ST4	557	441	475	292
Unpublished	2007	FOXP3	502	414	513	351
Unpublished	2008	P2RX7, TLR1	494	416	525	401
Unpublished	2010	CD14	387	341	406	321
Unpublished	2012	APOE	435	410	443	407
Unpublished	2013	IRGM, ISG15, NLRC5, NLRP3, NOD2	427	407	439	403

References

[1] Rossouw M, Nel HJ, Cooke GS, van Helden PD, Hoal EG (2003) Association between tuberculosis and a polymorphic NFκB binding site in the interferon γ gene. The lancet 361: 1871-1872.

[2] Hoal EG, Lewis LA, Jamieson SE, Tanzer F, Rossouw M, et al. (2004) SLC11A1 (NRAMP1) but not SLC11A2 (NRAMP2) polymorphisms are associated with susceptibility to tuberculosis in a high-incidence community in South Africa. The International Journal of Tuberculosis and Lung Disease 8: 1464-1471.

[3] Babb C, Keet EH, Helden PDv, Hoal EG (2007) SP110 polymorphisms are not associated with pulmonary tuberculosis in a South African population. Human Genetics 121: 521-522.

[4] Barreiro LB, Neyrolles O, Babb CL, van Helden PD, Gicquel B, et al. (2007) Length variation of DC-SIGN and l-SIGN neck-region has no impact on tuberculosis susceptibility. Human immunology 68: 106-112.

[5] Möller M (2007) Human genetic susceptibility to tuberculosis: the investigation of candidate genes influencing interferon gamma levels and other candidate genes affecting immunological pathways. Ph.D. thesis, Stellenbosch University. http://hdl.handle.net/10019.1/1264.

[6] Möller M, Kwiatkowski R, Nebel A, van Helden PD, Hoal EG, et al. (2007) Allelic variation in BTNL2 and susceptibility to tuberculosis in a South African population. Microbes and Infection 9: 522-528.

[7] Möller M, Nebel A, Kwiatkowski R, van Helden PD, Hoal EG, et al. (2007) Host susceptibility to tuberculosis: CARD15 polymorphisms in a South African population. Molecular and cellular probes 21: 148-151.

[8] Möller M, Nebel A, Valentonyte R, van Helden PD, Schreiber S, et al. (2009) Investigation of chromosome 17 candidate genes in susceptibility to TB in a South African population. Tuberculosis 89: 189-194.

[9] Möller M, Flachsbart F, Till A, Thye T, Horstmann RD, et al. (2010) A functional haplotype in the 3' untranslated region of TNFRSF1B is associated with tuberculosis in two African populations. American journal of respiratory and critical care medicine 181: 388-393.

[10] Möller M, Nebel A, Van Helden PD, Schreiber S, Hoal EG (2010) Analysis of eight genes modulating interferon gamma and human genetic susceptibility to tuberculosis: a case-control association study. BMC infectious diseases 10: 154.

[11] Adams LA, Möller M, Nebel A, Schreiber S, van der Merwe L, et al. (2011) Polymorphisms in MC3R promoter and CTSZ 3' UTR are associated with tuberculosis susceptibility. European Journal of Human Genetics 19: 676-681.

[12] Babb C (2007) Identification of candidate genes and testing for association with tuberculosis in humans. Ph.D. thesis, Stellenbosch University. http://hdl.handle.net/10019.1/21524.

[13] De Wit E (2009) Analysis of host determining factors in susceptibility to tuberculosis in the South African coloured population. Ph.D. thesis, Stellenbosch University. http://hdl.handle.net/10019.1/4584.

[14] Salie M (2010) Investigating candidate genes identified by genome-wide studies of granulomatous diseases in susceptibility to tuberculosis: ANXA11 and the CADM family. Master's thesis, Stellenbosch University. http://hdl.handle.net/10019.1/5472.

[15] Lucas L (2012) Toll-like receptor genes and their pathway: role in susceptibility to pulmonary tuberculosis in a South African population. Master's thesis, Stellenbosch University. http://hdl.handle.net/10019.1/20390.

[16] Wagman C (2012) Genetic studies on susceptibility to pulmonary tuberculosis mediated by MARCO, SP-D and CD14: molecules affecting uptake of mycobacterium tuberculosis into macrophages. Master's thesis, Stellenbosch University. http://hdl.handle.net/10019.1/20409.

[17] Bruiners N (2013) Investigating the Human-M. tuberculosis interactome to identify the host targets of ESAT-6 and other mycobacterial antigens. Ph.D. thesis, Stellenbosch University. http://hdl.handle.net/10019.1/71977.
