# Supplementary material for: Investigating the Role of Gene-Gene Interactions in TB Susceptibility
Source: PLoS One. 2015 Apr 28;10(4):e0123970. doi: 10.1371/journal.pone.0123970 (PMC4412713; doi:10.1371/journal.pone.0123970)
Supplement: S2 Table — P-values were calculated using logistic regression. (PDF) [file pone.0123970.s006.pdf]

			Age		Gender	
		**Sample size**	**Mean ± SD**	**P-value**	**Nr (Prop)**	**P-value**
**SAC candidate gene data**	TB cases	918	36.00 ± 12.79	< 0.0001	493 (0.54)	< 0.0001
	Controls	507	32.48 ± 10.69		122 (0.24)	
**SAC chip data**	TB cases	642	36.69 ± 11.50	< 0.0001	361 (0.56)	0.6210
	Controls	91	31.47 ± 4.09		45 (0.49)	
**Gambian data**	TB cases	1156			823 (0.72)	< 0.0001
	Controls	1206			574 (0.48)	
