# Supplementary material for: Investigating the Role of Gene-Gene Interactions in TB Susceptibility
Source: PLoS One. 2015 Apr 28;10(4):e0123970. doi: 10.1371/journal.pone.0123970 (PMC4412713; doi:10.1371/journal.pone.0123970)
Supplement: S3 Table — A summary listing web URLs, version information and important parameter settings of software used in this study. (PDF) [file pone.0123970.s007.pdf]

Program	Web URL	Version	Parameters
**PLINK**	http://pngu.mgh.harvard.edu/∼purcell/plink/	v1.07	*–indep-pairwise 50 10 0.1* was used for LD filtering; a custom version was used for the *fast-epistasis* tests
**Welcome Trust strand and build scripts**	http://www.well.ox.ac.uk/∼wrayner/strand/		The Affy-NSP-STY-b37.58-v4 strand and position files were used
**ADMIXTURE**	http://www.genetics.ucla.edu/software/admixture/	1.21	*K=5*
**EIGENSOFT**	http://genetics.med.harvard.edu/reich/Reich_Lab/Software.html	5.0.1	
**Biofilter**	http://ritchielab.psu.edu/software/biofilter-download	2.1.0	LOKI database was built on 5 Dec 2013
**R**	http://www.r-project.org	3.1.0	
**effects R package**	http://cran.r-project.org/web/packages/effects/index.html	3.0-1	The *effect()* function was used
**genetics R package**	http://cran.r-project.org/web/packages/genetics/index.html	1.3.8.1	The *HWE.exact()* and *LD* functions were used
**ggplot2 R package**	http://cran.r-project.org/web/packages/ggplot2/index.html	1.0.0	
**haplo.stats R package**	http://cran.r-project.org/web/packages/haplo.stats/index.html	1.6.8	The *group.bin()* function was used
