# Supplementary material for: Investigating the Role of Gene-Gene Interactions in TB Susceptibility
Source: PLoS One. 2015 Apr 28;10(4):e0123970. doi: 10.1371/journal.pone.0123970 (PMC4412713; doi:10.1371/journal.pone.0123970)
Supplement: S5 Table — This table provides a summary of each SNP’s individual minor allele frequency (MAF) and association with having TB. (PDF) [file pone.0123970.s009.pdf]

	Gene	Region	SAC			Gambian		
			**SNP**	**MAF**	**P-value**	**SNP**	**MAF**	**P-value**
**Model 1**	NRG1	8p12	rs16879814	0.22	0.8200	rs16879814	0.23	0.3952
	NRG3	10q23	rs11191757	0.33	0.1287	rs2224109	0.13	0.3365
**Model 2**	GRIK1	21q22	rs465555	0.49	0.4642	rs460583	0.42	0.1397
	GRIK3	1p34	rs3738085	0.39	0.8017	rs476894	0.40	0.4022
**Model 3**	SFTPD	10q22	rs1923537	0.28	0.8429			
	NOD2	16q12	rs748855	0.18	0.4041			
**Model 4**	IL23R	1p31	rs10489628	0.49	0.7888	rs10489628	0.12	0.1323
	ATG4C	1p31	rs11208029	0.11	0.3534	rs11208029	0.41	0.4644
**Model 5**	FUT8	14q23	rs17102844	0.05	0.3336	rs9323464	0.47	0.0140
	B4GALT1	9p13	rs12342831	0.25	0.8881	rs10758189	0.18	0.0358
**Model 6**	EXT1	8q24	rs6469713	0.02	0.8728			
	EXT2	11p11	rs903509	0.10	0.6867			
**Model 7**	ISG15	1p36	rs15842	0.14	0.0149			
	TLR8	Xp22	rs3761624	0.38	0.5738			
**Model 8**	NCAM2	21q21	rs8134735	0.04	0.7718	rs8132838	0.23	0.2967
	IRF8	16q24	rs8054065	0.47	0.1589	rs147968	0.18	0.1974
**Model 9**	ANK1	8p11	rs2102360	0.13	0.2941			
	ANK3	10q21	rs2393618	0.16	0.3319			
**Model 10**	NELL1	11p15	rs1377741	0.35	0.0798	rs1377741	0.39	0.1964
	NOS2	17q11	rs2297516	0.50	0.2571	rs2314809	0.46	0.3252
**Model 11**	CADM3	1q21	rs16841729	0.10	0.0562			
	SLC22A4	5q31	rs13179900	0.06	0.7064			
**Model 12**	ANK2	4q25	rs1354679	0.39	0.3830	rs1354679	0.31	0.7538
	ANK3	10q21	rs10821731	0.45	0.1023	rs10761481	0.43	0.8734
**Model 13**	NELL1	11p15	rs4614448	0.40	0.7614	rs4614448	0.43	0.0022
	CADM2	3p12	rs17024414	0.19	0.4179	rs17024876	0.46	0.2754
**Model 14**	NLRC5	16q13	rs289726	0.48	0.3474			
	IL12RB1	19p13	rs393548	0.19	0.4955			
**Model 15**	PLCB1	20p12	rs708914	0.42	0.7643	rs1703634	0.45	0.4542
	PLCE1	10q23	rs4918082	0.44	0.8491	rs4918082	0.48	0.7421
**Model 16**	C1QA	1p36	rs12033074	0.42	0.7034			
	TMEFF2	2q32	rs4077949	0.50	0.9403			
**Model 17**	NELL1	11p15	rs11025887	0.39	0.9114	rs12577018	0.47	0.2634
	CADM3	1q21	rs862991	0.45	0.6270	rs862991	0.31	0.6527
**Model 18**	PDE2A	11q13	rs171021	0.23	0.5357	rs3781931	0.16	0.9916
	PDE4B	1p31	rs536025	0.14	0.2809	rs17423910	0.05	0.4467
**Model 19**	CHST11	12q23	rs17036205	0.04	0.9026	rs17036205	0.22	0.2632
	CHSY3	5q23	rs32225	0.41	0.8596	rs244745	0.03	0.9337
**Model 20**	SLC22A4	5q31	rs2306772	0.07	0.6799	rs3792880	0.13	0.2623
	ALOX5	10q11	rs3740107	0.25	0.0164	rs3780909	0.49	0.3145
