# Supplementary material for: Investigating the Role of Gene-Gene Interactions in TB Susceptibility
Source: PLoS One. 2015 Apr 28;10(4):e0123970. doi: 10.1371/journal.pone.0123970 (PMC4412713; doi:10.1371/journal.pone.0123970)
Supplement: S6 Table — The genotypic model p-values, which were used to select the top 20 models, are presented in this table. The p-values of the corresponding allelic interaction models that achieved the smallest p-values are also shown. (PDF) [file pone.0123970.s010.pdf]

	Model	Genotypic	Allelic	
		P-value	Best model	P-value
1	NRG1_rs16879814 - NRG3_rs11191757	8.32×10^{-7}	dominant-recessive	8.80×10^{-6}
2	GRIK1_rs465555 - GRIK3_rs3738085	1.62×10^{-6}	additive-recessive	4.78×10^{-6}
3	SFTPD_rs1923537 - NOD2_rs748855	1.89×10^{-6}	recessive-additive	5.57×10^{-7}
4	IL23R_rs10489628 - ATG4C_rs11208029	2.18×10^{-6}	dominant-dominant	8.53×10^{-6}
5	FUT8_rs17102844 - B4GALT1_rs12342831	2.54×10^{-6}	dominant-recessive	4.18×10^{-7}
6	EXT1_rs6469713 - EXT2_rs903509	2.67×10^{-6}	additive-additive	2.49×10^{-6}
7	ISG15_rs15842 - TLR8_rs3761624	6.23×10^{-6}	dominant-recessive	1.15×10^{-6}
8	NCAM2_rs8134735 - IRF8_rs8054065	8.06×10^{-6}	dominant-dominant	1.32×10^{-6}
9	ANK1_rs2102360 - ANK3_rs2393618	8.96×10^{-6}	dominant-additive	3.20×10^{-6}
10	NELL1_rs1377741 - NOS2_rs2297516	8.98×10^{-6}	additive-additive	1.09×10^{-6}
11	CADM3_rs16841729 - SLC22A4_rs13179900	9.62×10^{-6}	dominant-dominant	1.38×10^{-5}
12	ANK2_rs1354679 - ANK3_rs10821731	1.14×10^{-5}	recessive-dominant	3.79×10^{-5}
13	NELL1_rs4614448 - CADM2_rs17024414	1.14×10^{-5}	recessive-dominant	1.20×10^{-4}
14	NLRC5_rs289726 - IL12RB1_rs393548	1.16×10^{-5}	recessive-dominant	1.26×10^{-4}
15	PLCB1_rs708914 - PLCE1_rs4918082	1.20×10^{-5}	recessive-dominant	7.17×10^{-4}
16	C1QA_rs12033074 - TMEFF2_rs4077949	1.25×10^{-5}	recessive-dominant	6.91×10^{-6}
17	NELL1_rs11025887 - CADM3_rs862991	1.26×10^{-5}	recessive-dominant	1.53×10^{-6}
18	PDE2A_rs171021 - PDE4B_rs536025	1.29×10^{-5}	recessive-additive	7.80×10^{-7}
19	CHST11_rs17036205 - CHSY3_rs32225	1.34×10^{-5}	dominant-dominant	1.18×10^{-3}
20	SLC22A4_rs2306772 - ALOX5_rs3740107	1.37×10^{-5}	dominant-dominant	8.29×10^{-4}
